# Modulation of gut microbiota, blood metabolites, and disease resistance by dietary β-glucan in rainbow trout (*Oncorhynchus mykiss*)

**DOI:** 10.1186/s42523-022-00209-5

**Published:** 2022-11-20

**Authors:** Simon Menanteau-Ledouble, Jakob Skov, Mie Bech Lukassen, Ulrike Rolle-Kampczyk, Sven-Bastiaan Haange, Inger Dalsgaard, Martin von Bergen, Jeppe Lund Nielsen

**Affiliations:** 1 Department of Chemistry and Bioscience, Aalborg University, Fredrik Bajers Vej 7H, 9220 Aalborg East, Denmark; 2 Department of Veterinary and Animal Sciences, University of Copenhagen, Grønnegårdsvej 15, 1870 Frederiksberg C, Denmark; 3 National Institute of Aquatic Resources, Technical University of Denmark, Kemitorvet, 2800 Kongens Lyngby, Denmark; 4 Department of Molecular Systems Biology, Helmholtz Centre for Environmental Research, UFZ, Permoserstr. 15, 04318 Leipzig, Germany; 5 German Centre for Integrative Biodiversity Research, (iDiv) Halle-Jena-Leipzig, Puschstraße 4, 04103 Leipzig, Germany; 6 Institute of Biochemistry, Faculty of Life Sciences, University of Leipzig, Brüderstraße 34, 04103 Leipzig, Germany

**Keywords:** *Oncorhynchus mykiss*, Gut microbiota, β-glucan, Prebiotic, Metabolite response

## Abstract

**Background:**

Prebiotics are known to have a positive impact on fish health and growth rate, and β-glucans are among the most used prebiotics on the market. In this study, rainbow trout (*Oncorhynchus mykiss*) were treated with a β-1,3;1,6-glucan dietary supplement (at a dose of 0 g, 1 g, 10 g, and 50 g β-glucan per kg of feed). After 6 weeks, the effect of the β-glucan was evaluated by determining the changes in the microbiota and the blood serum metabolites in the fish. The impact of β-glucan on the immune system was evaluated through a challenge experiment with the bacterial fish pathogen *Yersinia ruckeri.*

**Results:**

The microbiota showed a significant change in terms of composition following β-glucan treatment, notably an increase in the relative abundance of members of the genus *Aurantimicrobium*, associated with a decreased abundance of the genera *Carnobacterium* and *Deefgea*. Furthermore, analysis of more than 200 metabolites revealed that the relative levels of 53 metabolites, in particular compounds related to phosphatidylcholines, were up- or downregulated in response to the dietary supplementation, this included the amino acid alanine that was significantly upregulated in the fish that had received the highest dose of β-glucan. Meanwhile, no strong effect could be detected on the resistance of the fish to the bacterial infection.

**Conclusions:**

The present study illustrates the ability of β-glucans to modify the gut microbiota of fish, resulting in alteration of the metabolome and affecting fish health through the lipidome of rainbow trout.

**Supplementary Information:**

The online version contains supplementary material available at 10.1186/s42523-022-00209-5.

## Background

Aquaculture has undergone a fast and sustained expansion and intensification over the last several decades [1]. Meanwhile, antibiotic-resistant bacteria have continued to rise in prevalence and have become one of the main concerns in terms of global health [2]. Under these circumstances, there is a strong need to develop alternatives to the use of antibiotics, including in aquaculture settings. Prophylactics in particular are of great interest, and multiple products are being commercialized aimed at protecting fish stocks by stimulating the animals' immune system or otherwise pre-empting bacterial infections. Probiotics, for example, are benign bacteria specifically selected and given to the fish to enrich their microbiota in a beneficial fashion [3]. Probiotics have shown protective effects in fish [4, 5], notably in rainbow trout (*Oncorhynchus mykiss*), where their immunostimulatory properties are well established [6]. Similarly, phytogenic feed complements have been shown to protect rainbow trout against infections by *Aeromonas salmonicida* [7–9].

In addition to probiotics, prebiotics constitute another commonly applied type of immunostimulatory substance. The term refers to substances that are not metabolised to a significant extent but instead manipulate a host’s microbiota in beneficial ways [10]. This definition is often refined to only include fibres and non-digestible mono-, oligo- or polysaccharides in particular, to the exclusion of other potential molecules, with a focus on the intestinal microbiota, although these restrictions are not recognised by the International Scientific Association for Prebiotics [10]. In addition to their effect on the intestinal microbiota and the digestion, prebiotics have been documented to act as immunostimulants, enhancing intestinal functions and favourably impacting host growth [11–13]. A number of prebiotics are available for use in aquaculture, and β-glucans are some of most widely used prebiotics [14]. β-Glucans can be found in bacteria, plants, algae and fungi, where they constitute a diverse group of linear polysaccharides linked by either β(1,3) or β(1,4)-glycosidic bonds with side branches connected to the principal chain by β(1,2) or (1,6)-glucopyranosyl substituents [14–17]. β-Glucans are known to stimulate the phagocytic activity and superoxide production of macrophages extracted from the anterior kidney of Atlantic salmon (*Salmo salar*) while increasing lysozyme activity within that organ [18]. Injection of Atlantic salmon with β-glucans can induce the overexpression of several markers of inflammation in the spleen, notably interleukin-1β and tumour necrosis factor α [19]. Furthermore, oral and bath administration of β-glucans to rainbow trout has been reported to significantly modulate immune gene expression in the anterior kidney, skin, and gill tissue, respectively [20, 21]. Additionally, β-glucans have been found to improve the farming performance of *O. mykiss*, being associated with significantly higher specific growth rate and significantly reduced feed conversion ratio in juvenile *O. mykiss* [22]*.* However, reports of the efficacy of β-glucans have been inconsistent with several studies finding limited or no effects [23], and the mechanisms through which β-glucans exert their effects are still largely unknown. For example, the exact receptors through which β-glucans are recognized by the teleost immune system remain to be elucidated: Several cellular receptors have been associated with β-glucan recognition in mammals, notably the Fc-g receptor and the complement receptor 3 [24], the homologs of which are present in teleosts, as well as dectin-1, a member of the lectin superfamily, which is present in mammals, but not in teleosts [24, 25]. However, a recent study on β-glucan-activated macrophages in carp has allowed the identification of several genes encoding C-type lectin domains that were upregulated in the presence of β-glucan and represent possible receptors for β-glucan, acting as the fish equivalent to dectin-1 [26].

In addition to these immunostimulatory effects, several studies have shown that the gut microbiota in the fish is altered by the diet. For example, replacement of animal-based proteins by plant-based proteins resulted in higher abundances of members of the genera *Streptococcus*, *Leuconostoc,* and *Weissella* in the fish's intestine [27]. Interestingly, Ingerslev et al. also investigated the ability of the probiotic bacterium *Pediococcus acidilactici* to assist in the digestion of the plant diet but found that this addition did not significantly alter the composition of the microbiota or the performance of the fish compared to the same diet without the probiont. These later findings were in disagreement with those of other studies that showed that addition of *P. acidilactici* to the diet of *O. mykiss* did result in alteration of the intestinal microbiota [28]. Equally interestingly, another study also reported the effect of these probiotics on the fish's susceptibility to infection by *Yersinia ruckeri,* but only found limited effects [29].

Concerning β-glucans, it has further been reported that administration of this prebiotic substance also resulted in alteration of the fish's microbiota [30, 31]. Bacterial richness and diversity have been shown to be affected in the gut of fish fed β-glucan-supplemented diets, and the main responses were shown to occur through increased levels of the phyla *Proteobacteria*, *Firmicutes*, *Fusobacteria* as well as hitherto unidentified and non-culturable groups of bacteria [30]. The proportion of the lactic acid bacteria population has also been observed to decrease in the gut of *Cyprinus carpio* fed a diet supplemented with β-(1,3)(1,6)-D-glucan [30]. Moreover, it has more recently been shown that the gut microbiota could influence host health through alterations in the levels of various metabolites in the gut as well as in the blood serum of the host [32]. Cholesterol, fatty acid, and carbohydrate metabolisms have been reported to change when the fish gut microbiota shifted [32–34]. It is therefore likely that a change in the microbiota induced by the administration of β-glucans could have significant health benefits.

Based on this data, we hypothesized that supplementation of the fish's diet with β-glucan would alter the composition of the fish's intestinal microbiota, and that it would correlate with changes in the fish’s metabolism, including in the serum, resulting in changes in the fish immune status and susceptibility to infection. To test these hypotheses, a well-characterized β-glucan was investigated in three different concentrations (0.1%, 1.0% and 5.0%). Microbial populations were characterized by a high throughput 16S rRNA gene amplicon sequencing, while the metabolites were analysed by a LC-MS-MS method. Finally, the effect on fish health was evaluated by a challenge experiment with exposure to *Y. ruckeri*.

## Methods

### Fish and rearing conditions

Female rainbow trout (*Oncorhynchus mykiss*) (*n* = 550) were hatched from disinfected eggs (30 min in 50 mg∙L^−1^ iodine) and reared under specific pathogen-free conditions (Bornholm Salmon) to an approximate individual size of 10 g. The fish were transferred to 120 L glass aquaria (eight aquaria with 60 fish each plus two aquaria with 35 fish each, Additional file 1: Table S1) containing aerated municipal tap water at a mean temperature of 14.8 ± 0.6 °C (SD), internal biofilters (20 L∙min^−1^, EHEIM), and a 12 h light/12 h dark cycle. The fish were acclimated to the laboratory conditions and a diet of INICIO 917 (BioMar A/S) at a rate of 1% biomass day^−1^ for one week, followed by three weeks of acclimatization to the control diet (i.e. rapeseed oil-supplemented INICIO 917; 1% biomass·day^−1^) prior to onset of experimental feeding.

### Experimental feed and feeding

A commercial pelleted (1.5 mm) trout feed (INICIO 917, BioMar A/S) based on a mixture of fish and plant protein and containing 47% crude protein, 20% crude lipid, 18% carbohydrates (NFE), 1.2% fibre, 8.5% ash, and 1.1% phosphorus was used for preparation of the diets. A β-1,3;1,6-glucan (purity: 81.6%; mean particle size: 37.7 µm; Additional file 1: Table S2) purified from yeast (*Saccharomyces cerevisiae*) (Biorigin, Brazil) was supplemented to this diet at varying doses: 0 g, 1 g, 10 g, and 50 g β-glucan were added to 1 kg of INICIO 917, respectively, during continuous mixing and sealed to the pellets by spraying with 30 mL organic, cold-pressed rapeseed oil (Gyldenmark), resulting in a control diet without β-glucan and three experimental diets of 0.1%, 1.0%, and 5.0% Wt/Wt β-glucan.

Ten representative randomly selected fish were weighed and measured at the time of allocation and then at week 3, 6, 8, 9, and 10 afterwards and the Fulton’s condition factor was calculated [35].

### Blood and gut sampling

For the metabolite and microbiota analyses, 10 fish from the control group were collected on day 0 and 10 fish from each group (control, 0.1, 1.0, 5.0% β-glucan) at week 6. Fish were euthanized by immersion in an overdose (200 mg∙L^−1^) of MS-222 (cat no. A5040, Sigma-Aldrich). The tail was cut, and blood was sampled from the *vena caudalis* using Na-heparinised 25 mL and 50 mL capillary pipettes (Hirschmann Laborgeräte, Germany). Blood samples were centrifuged at 3000 g for 10 min at 4 °C, and serum was isolated and stored at -80 °C. The intestine was aseptically sampled and immediately immersed in RNA*later*® (Sigma-Aldrich) and transferred to − 20 °C, as recommended elsewhere [36].

### DNA extraction and amplicon preparation

Stools were harvested from the intestines, taking care not to damage the lining of the intestine and genomic DNA was extracted using the DNeasy Blood and Tissue kit (Qiagen) according to the manufacturer’s recommendations. The bacterial community profiling in the gut of the fish was conducted by amplicon sequencing of the V1-3 region of the 16S rRNA gene using an Illumina MiSeq platform as described elsewhere [37].

The extracted DNA and amplicons were quality-controlled using the Tapestation 2200 and Genomic DNA ScreenTape (Agilent) before being quantified with a Quant-iT HS DNA Assay (Thermo Fisher Scientific) on an Infinite M1000 PRO (Tecan). The amplicons were pooled in equimolar concentrations and sequenced on a MiSeq (Illumina), using a MiSeq Reagent kit v3 (2 × 300 PE).

### Metabolite measurements

The metabolite response to the dietary β-glucan supplementation was determined using a targeted metabolic approach.

Metabolome analyses were carried out with the Absolute*IDQ*® p180 Kit (Biocrates Life Science AG, Austria). The Kit identifies and quantifies 188 metabolites from 5 compound classes (acyl carnitines, amino acids, glycerophospho- and sphingolipids, biogenic amines and hexoses).

The amino acids and biogenic amines were analysed by liquid chromatography (LC)–mass spectrometry (MS) (LC–MS/MS). The substance classes of acylcarnitines, phosphatidylcholines (including lysophosphatidylcholines), sphingomyelines and hexoses were analysed by flow injection analysis (FIA)-MS/MS measurements (FIA-MS/MS). Serum samples (10 µL) were mixed with isotopically labelled internal standard in a multititer plate and dried under nitrogen (nitrogen evaporator 96 well plate, VLM GmbH, Bielefeld, Germany). Afterwards the metabolites were derivatized with phenylisothiocyanate (PITC) 5% for 20 min at room temperature and subsequently dried for 30 min under nitrogen flow. For extraction first 300 µL of extraction solvent (5 mM ammonium acetate in methanol) were added and incubated with shaking at 450 rpm (Thermomixer comfort Eppendorf, Hamburg, Germany) for 30 min at room temperature, followed by filtration by centrifugation (Sigma, Taufkirchen) for 2 min at 5009 g. Subsequently, 200 µL were removed from the filtrate, transferred to a fresh multititer deep well plate and diluted with 200 µL of water for LC–MS analysis of biogenic amines and amino acids. To the remaining 100 µL from the filtrate 500 µL of MS running solvent were added for FIA-MS/MS. Both types of measurements were performed on a QTRAP mass spectrometer applying electrospray ionization (ESI) (ABI Sciex API5500Q-TRAP). The MS was coupled to an ultra-performance liquid chromatography (UPLC) (Waters Acquity, Waters Corporation, Milford, USA). In case of LC–MS the metabolites were separated by a hyphenated reverse phase column (Agilent, Zorbax Eclipse XDB C18, 3.0 9 100 mm, 3.5 µm; Agilent Waldbronn, Germany) preceded with a precolumn (Security Guard, Phenomenex, C18, 4 9 3 mm; Phenomenex, Aschaffenburg, Germany) applying a gradient of solvent A (formic acid 0.2% in water) and solvent B (formic acid 0.2% in acetonitrile) over 7.3 min (0.5 min 0% B, 5 min 70% B, 0.3 min 70% B, 2 min 0% B) at a flow rate of 500 µL/min. Oven temperature was 50 °C. For LC–MS analysis 10 µL, and for FIA 2 × 20 µL were subjected for measurements in positive and negative mode, respectively. Identification and quantification were achieved by multiple reaction monitoring (MRM) standardized by applying spiked-in isotopically labelled standards in positive and negative mode, respectively. For calibration, a calibrator mix consisting of seven different concentrations was used. Quality controls deriving from lyophilized human plasma samples were included for 3 different concentration levels. For FIA an isocratic method was used (100% organic running solvent) with varying flow conditions (0 min, 30 µL∙min^−1^; 1.6 min 30 µL∙min^−1^; 2.4 min, 200 µL∙min^−1^; 2.8 min, 200 µL∙min^−1^; 3 min 30 µL∙min^−1^), and the MS settings were as follows: scan time 0.5 s, IS voltage for positive mode 5500 V, for negative mode − 4500 V, source temperature 200 °C, nitrogen as collision gas medium; the corresponding parameters for LC–MS were: scan time 0.5 s, source temperature 500 °C, nitrogen as collision gas medium). All reagents used in the processing and analysis were of LC–MS grade, unless otherwise stated. Milli-Q Water ultrapure was used fresh after being prepared by the high-purity water system by Merck KGaA (Darmstadt, Germany). LC–MS grade acetonitrile (1.00029.2500) and methanol (1.000971.500), as well as pyridine for analysis (1.09728.0100) and formic acid (98–100%; 1.000263.1000) were purchased by Merck KGaA. PITC (P10034-10) and ammonium acetate (81.7838-50) were purchased by Sigma Aldrich Chemie GmbH (Steinheim, Germany). For the LC–MS assay the data analysis and calculation of the concentrations was performed in the Analyst Software (1.6.2.) The obtained LC results are then imported as.rdb file into MetIDQ software and can be assessed together with results from the FIA assay. The software is an integrated part of the Kit. This streamlines data analysis by automated calculation of metabolite concentrations providing quality measures and quantification.

### Experimental infection and disease assessment

After 6 weeks of β-glucan-supplemented feeding, 40 fish (mean (SD) mass = 21.5 (5.8) g; *n* = 40) in duplicate from each experimental group were experimentally infected by bath exposure to 1.6 × 10^7^ CFU∙mL^−1^ of live *Y. ruckeri* O1 biotype 2 (100415-1/4) for 9 h. Thirty-five fish fed the control diet were exposed in duplicate to pure water without *Y. ruckeri* for 9 h and served as uninfected controls. Subsequent to experimental infection, fish were monitored for 28 days for development of clinical signs of disease. Humane endpoints were defined as darkened skin combined with apathy and/or early signs of loss of balance, at which point fish were euthanized by immersion in an overdose (200 mg∙L^−1^) of ethyl 3-aminobenzoate methanesulfonate (syn. MS-222) (cat no. A5040, Sigma-Aldrich) and recorded as mortality. In order to confirm *Y. ruckeri* as the cause of morbidity, and to follow the clearance of the bacterium following the experimental infection, swab samples of head kidney were taken according to established protocols [38] and cultured on 5% blood agar (SSI Diagnostica) at 20 °C for 48 h from fish reaching humane endpoints and from 10, 20 and 30 fish, randomly selected on day 3, 14, 21, and 28 post infection (p.i.), respectively. Bacterial clearance was assessed based on the presence or absence of *Y. ruckeri* among the experimentally infected fish over time.

### Bioinformatic and statistical analyses

Raw sequencing data were converted into amplicon sequencing variants using the AmpProc pipeline version 5.1 (https://github.com/eyashiro/AmpProc). The pipeline was executed in paired-end mode for the V1–3 amplicon and the resulting sequences were matched against the Microbes of Activated Sludge and Anaerobic Digesters (MIDAS v4.8.1) database [39].

Principal component analysis (PCA), constrained ordination through redundancy analysis (RDA), heatmap, alpha diversity index (Chao1) and observed species richness were analysed, using R (version 4.0.3), RStudio (version 2022.02.3), and the R packages Ampvis [40], Phyloseq [41], and ggplot2 [42]. Chao1 indices were tested for normality using Shapiro test and then compared using paired t-tests. Alteration in the composition of the microbiota were first investigated using PCA (Additional file 1: Fig. S3). Then, the effect of the diet was investigated by performing RDA on sequence reads numbers, using the diet as the constraint and permutation tests were performed to confirm the significance of this effect based on the RDA. Differences in the metabolite response for the different diet treatments were analysed by pairwise Wilcoxon rank sum tests using the Bonferroni correction using the Phyloseq package.

## Results

### Richness and diversity

**Fig. 1:**
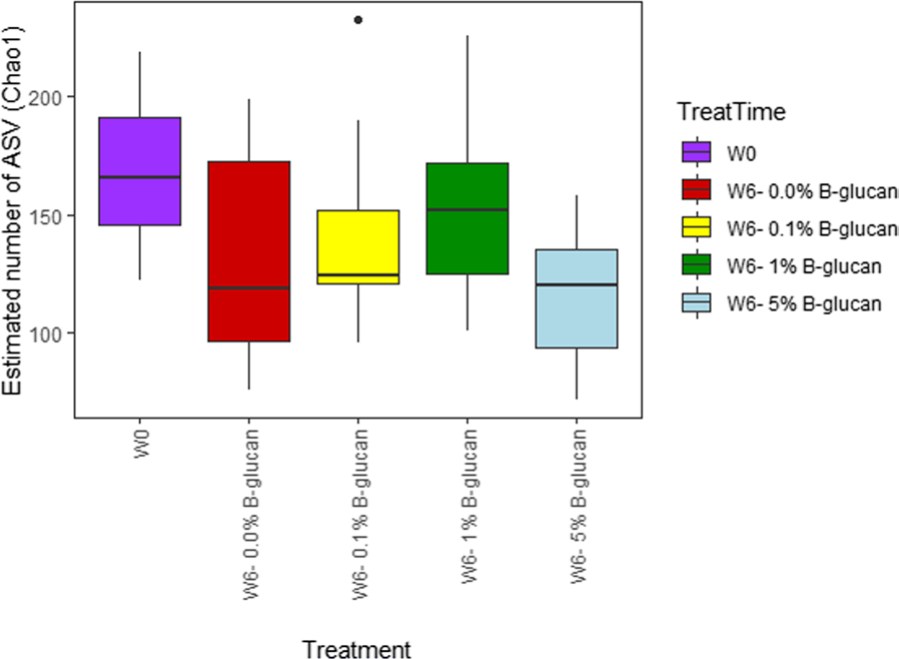
Alpha diversity measured in Chao1. W0: Diversity for control week 0. W6: Diversity for control week 6. 0.1%: Diversity for microbiota treated with 0.1% β-glucan. 1.0%: Diversity for microbiota treated with 1.0% β-glucan. 5.0%: Diversity for microbiota treated with 5.0% β-glucan

The observed species richness appeared to be reduced as the fish aged, dropping from an average Chao1 index of 169 at week 0 to an average of 136 at week 6, even if this difference were not significant. There was no obvious pattern between the fish at week 6 with the highest diversity being calculated in the 0.1% and 1.0% β-glucan treatment while the lowest diversity was found in the fish that had received 5.0% β-glucan treatment (Fig. 1). However, this was again not statistically significant (one-way ANOVA *p* > 0.23).

### Differences in microbial communities

In all sampled fish, the 3 most common phyla were *Actinobacteriota*, *Proteobacteria*, and *Firmicutes*, accounting for over 91% of the sequences detected in the fish at week 0 and over 95% of the sequences in the fish at week 6 (Additional file 1: Fig. S4). Of these three phyla, *Firmicutes* were the most common in the fish in the control groups with *Actinobacteriota* being the least represented phylum in the fish in the control group at week 6. On the contrary, the phylum *Actinobacteriota* was the most common in the fish that had received the β-glucan with *Firmicutes* being the least represented among these 3 major phyla; its relative abundance decreasing as the dose of β-glucan increased.

**Fig. 2:**
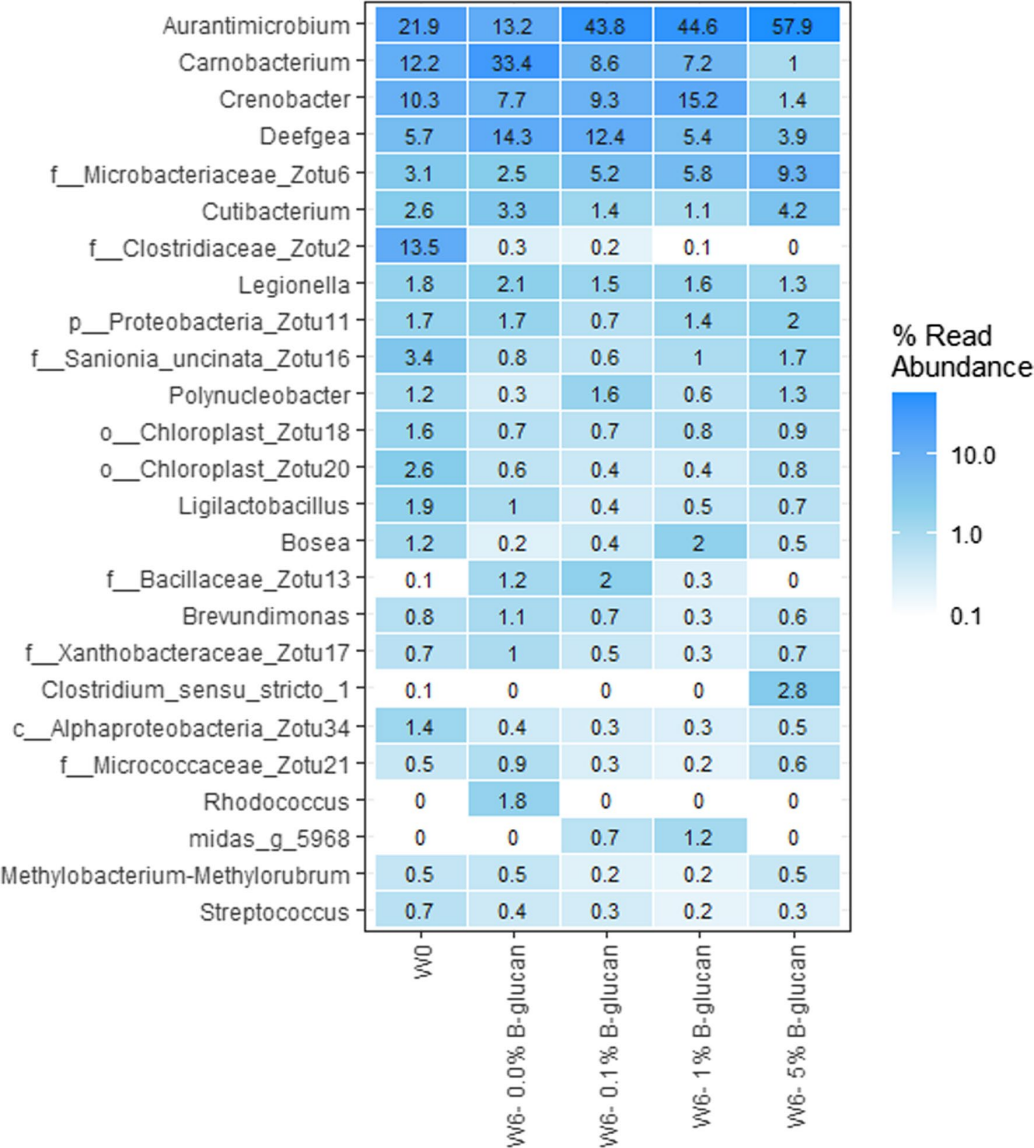
Heatmap showing the relative abundance in term of sequence reads for the 20 most abundant bacterial genera in the intestines of the fish receiving the different concentration of β-glucan and the control groups. Individual numbers on the heatmap indicate the relative abundance of each genus

At the genus level, control fish sampled at weeks 0 and 6 showed differences in terms of microbial composition with the most dominant genera at week 0 being *Aurantimicrobium* sp., followed by *Carnobacterium* sp., with this trend being inverted at week 6 when *Carnobacterium* was the most relatively abundant genus and *Deefgea* and *Aurantimicrobium* being similar in abundance (Fig. 2). Moreover, members of the family *Clostridiaceae* accounted for 13.5% of the sequences identified in the fish at week 0 but they were almost absent in all the fish at week 6.

Interestingly, addition of the β-glucan restored the prominence of *Aurantimicrobium* sp. with the higher doses of the prebiotic being associated with higher relative abundance of this genus. Conversely, supplementation with β-glucan was associated with reduced relative abundances of *Carnobacterium* sp. and *Deefgea* sp. with, once again, this effect being more marked as the dose of the prebiotic increased. Finally, the pattern of *Crenobacter* sp. was more complex with its relative abundance appearing to increase with higher doses of β-glucan, all the way to the highest dose of the prebiotic (5.0%), at which point the relative abundance of this genera was the lowest of any treatment.

**Fig. 3:**
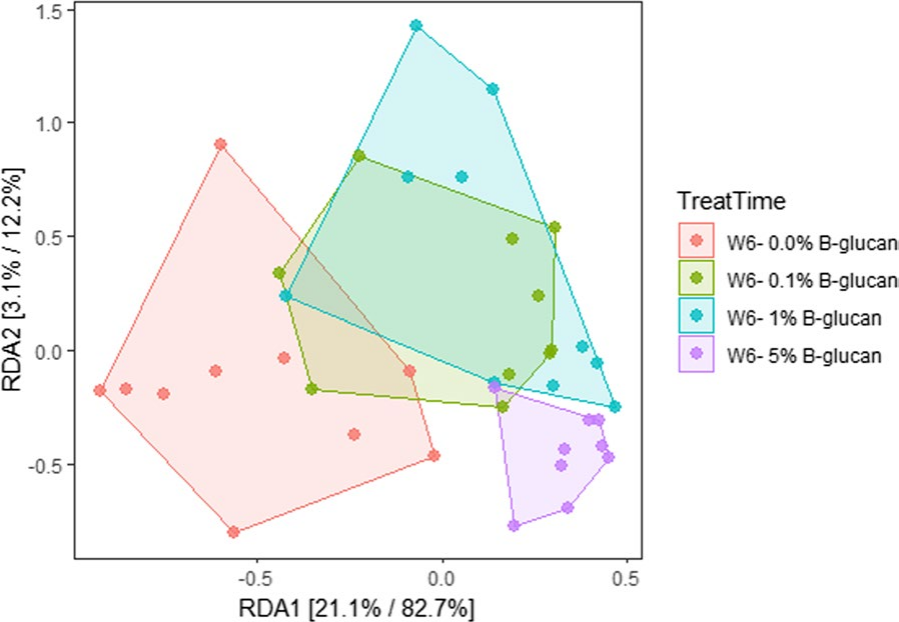
Redundancy analysis plot illustrating the composition of the microbiota in the intestine of the fish at week 6, constrained based on the concentration of β-glucan in the diet. Percentages in the axis indicate the percentage of the total variation explained by the treatment group; followed by the effect of this axis on the total variation between samples

The microbial communities for the three different β-glucan treatments and the control group for week 6 were significantly different (permutation test, *p* < 0.001) (Fig. 3). The increased proportion of β-glucan in the treatments was associated with a rightward shift of the microbial communities in the ordination plot with the microbiota associated with the 0.1% and 1.0% β-glucan-supplemented diets largely overlapping each other and being located between the control and the 5.0% β-glucan. The points in the 5.0% β-glucan were clearly separated from the control and were more tightly grouped together. Interestingly, the microbiotas of the fish at week 0 appeared more dissimilar to that of the control at week 6 than to the treated fish (data not shown).

### Fish and metabolic response

Administration of the beta-glucan did not results in changes in the weight gain (Additional file 1: Table S5 and Additional file 1: Fig. S6) or Fulton’s condition factor (Additional file 1: Table S5 and Additional file 1: Fig. S7).

**Fig. 4:**
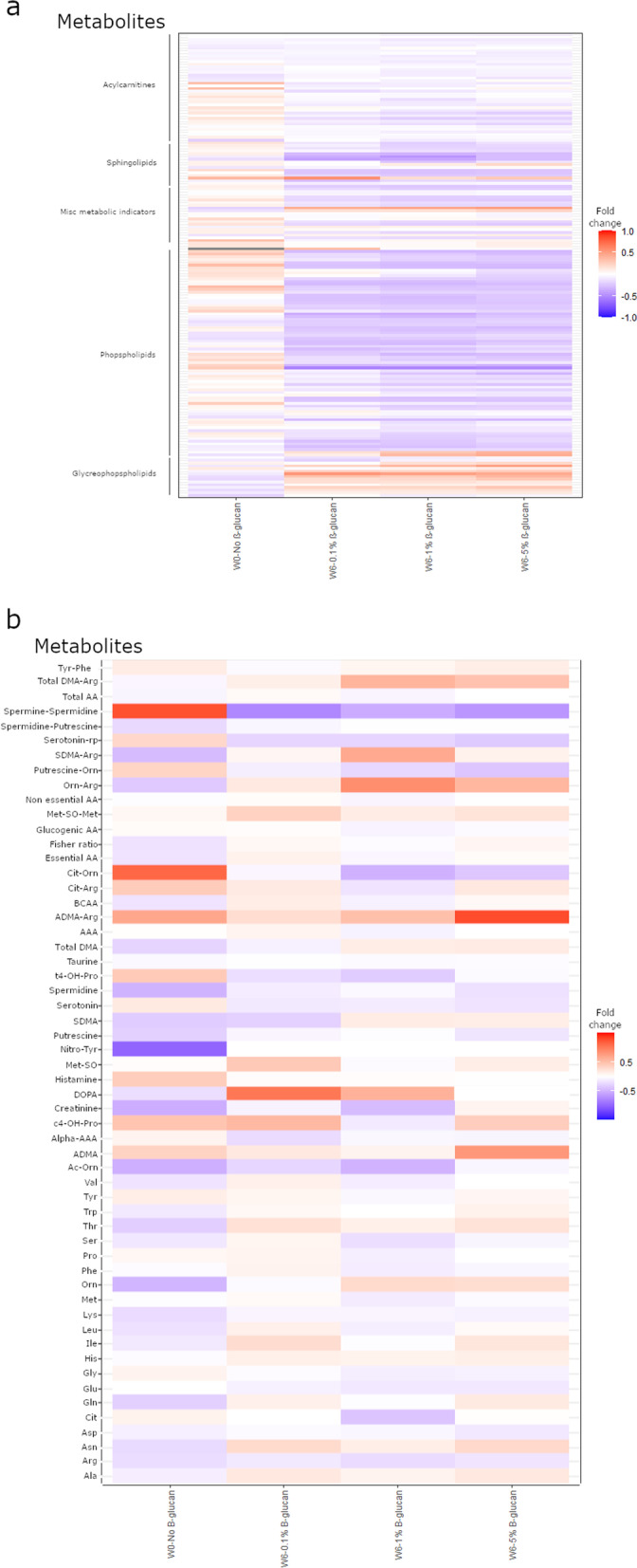
Heatmap showing the relative abundance of the serum’s metabolites compared to the control at week 6 (W6- 0.0% β-glucan) using pairwise Wilcoxon rank sum tests. Only samples that are significantly different from the control are shown and the colour of the square indicates the intensity of the change.** A**: LC samples.** B**: FIA samples

A diverse repertoire of metabolites was measured in the blood serum of the fish in order to assess the effect of dietary β-glucan exposure. More than 200 compounds were analysed and their levels compared between the different β-glucan treatments (Additional file 1: Table S8). All compounds with a statistically significant response (as shown by pairwise Wilcoxon rank sum tests) following exposure to the various β-glucan levels are shown in Additional file 1: Table S9, and the abundance of the compounds, in fold changes compared to the week 6 controls, are graphically depicted in Fig. 4. Of the 200 compounds investigated, 3 showed a significant (*p* < 0.05) downregulation among all three concentrations of β-glucan: 2 phosphatidylcholines with acyl-alkyl residue sums C36:4, C36:5 and a hydroxysphingomyelin with acyl residue sum C18:1. Only the ratio of lysophosphatidylcholine per phosphatidylcholines was higher in all 3 concentrations of β-glucan. Most metabolites (39) only showed only significant difference relative to the control for the high β-glucan addition (36 downregulated and 3 upregulated, including alanine, the only amino-acid differentially regulated). Meanwhile, one phosphatidylcholines with acyl-alkyl residue sums C32:1 was downregulated at the two lower β-glucan concentrations, and 3 compounds were only significantly different (all downregulated) at the lowest β-glucan concentration. None of the metabolites that were significantly altered due to β-glucan at two or all three concentrations showed conflicting up- or downregulations. The general pattern showed a change over time, with the administration of β-glucans further amplifying that change (Fig. 4a). For example, phospholipids were found to be less abundant in the controls at week 6 than at week 0 and this abundance was further reduced in a dose-dependent manner upon administration of the β-glucan. A similar pattern was observed for the plasma acylcarnitines with a carbon number between 14 and 18, but this trend was not obvious for acylcarnitines of different length. Conversely, glycerophospholipids were generally more abundant at week 6 than at week 0 and more abundant in the sera of the fish that had received the β-glucans compared to the controls.

Regarding amino acids, a general trend was observed in which the concentration was higher in the control at week 6 than at the start of the experiment and higher in the fish that had received the β-glucan compared to the control (Fig. 4b). However, this was not a direct dose response as the response of fish that had received the 0.1% β-glucan dose was generally lower than the controls and the other treatment groups. Moreover, only Alanine was significantly upregulated compared to the control and only at the highest concentration of β-glucan (*p* < 0.05). Meanwhile, biogenic amines showed a similar pattern in fish that had received the 0.1% dose similarly displaying lower concentration of these metabolites, however, none of these changes were statistically significant. Finally, the compound nitrotyrosine was detectable in 8 out of 30 (27%) of the fish that had received the β-glucan but only in one of the fish that had not (10%; *p* = 0.08).

### Bacterial clearance post infection

The experimental *Y. ruckeri* infection was mild and for most fish did not result in clinical signs severe enough to warrant euthanasia and therefore did not affect the experimental survival rate. Only two fish were euthanized due to disease; one fish treated with 1.0% β-glucan on day 9 p.i., and one fish treated with 5.0% β-glucan on day 14 p.i. (Additional file 1: Fig. S10). Both fish tested positive for *Y. ruckeri* by head kidney swabs cultured on blood agar. Uninfected controls showed 100% survival.

**Fig. 5:**
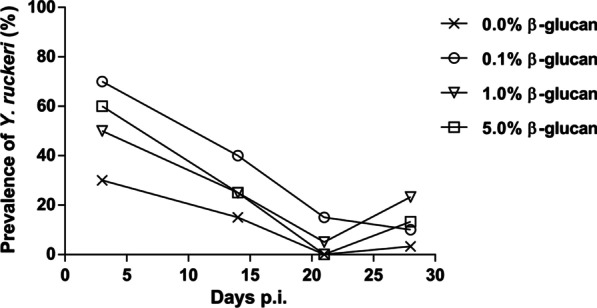
Clearance of *Y. ruckeri* among experimentally infected fish shown as the prevalence of *Y. ruckeri* among the groups. Sampling size (*n*) was 10, 20, 20, 30 fish per group at 3, 14, 21, and 28 days p.i., respectively

All β-glucan-treated fish showed a markedly higher *Y. ruckeri* prevalence on day 3 p.i. (Fig. 5) as determined by culture of kidney samples on blood agar plates, although this difference was not statistically significant at any time point. At day 14 p.i., bacterial prevalence had decreased in all groups, still being higher in β-glucan-treated fish compared to controls. The lowest bacterial prevalence was observed on days 21 and 28 p.i.

## Discussion

The present study was conducted to investigate the effect of supplementing the diet of rainbow trout with a β-1,3;1,6-glucan prebiotic. Several types of β-glucans are already used as prebiotics in aquaculture, but only a few studies have investigated their effects and mode of actions. Moreover, most of the studies conducted on β-glucans have focused on uncharacterized compounds, and thus the observed effects most frequently derive from a broad mixture of compounds [31, 43].

In the present study, fish were fed three different amounts of β-glucan (0.1%, 1.0%, and 5.0%) to study the effect on (i) survival after introduction of *Y. ruckeri*, (ii) the microbial community composition in the gut, and the (iii) metabolites in the blood serum of the fish.

A clear impact of β-glucans on the fish gut microbiota was observed, supporting previous observations in carp [30, 31] and in Atlantic cod [31]. Identification of the up- and downregulated microorganisms constitutes an important step in understanding how the β-glucans influence the gut microbiota, and how this change in the microbiome can affect the host by altering the nutrients, immune system, and/or compounds taken up through the epithelial barrier [44]. No statistically significant difference was observed in the species diversity in the intestines of the fish that had received the various treatment, although the lowest concentration of β-glucan was associated with the lowest Chao1 index (Fig. 1). In carp, it has been reported that diet supplementation with β-glucan reduced the diversity of the gut microbiota [30], although this finding was not universal and is contradicted by another study that reported an increase in species richness following supplementation of β-glucan to the diet of *C. carpio* [43]. This is one example of the inconsistencies associated with the effects of β-glucans and could plausibly be explained by the fish harbouring different microbiota that are therefore affected in different manners. The factors responsible for shaping the gut microbiota are complex and still largely remain to be investigated but appear to depend primarily on the feed, fish species, sex, and environment [44–49]. However, to a large extent, the composition of the gut microbiota still resembled what has previously been found in these studies. The most abundant species included *Firmicutes*, *Proteobacteria,* and *Actinobacteria* which is a common finding in fish [32, 50, 51]. The microbial composition of the trout used in this study evolved over time, and the samples obtained at week 0 and week 6 were significantly different, which reflects the effect of age and the importance of involving proper controls for comparability [52]. The most dominant phyla in the β-glucan-treated groups were primarily the same phyla as the control, but at altered relative abundance (*Actinobacteria* > *Proteobacteria* > *Firmicutes*). The relative abundance of *Actinobacteria* increased extensively in the gut of β-glucan-treated fish. However, relative abundance of *Firmicutes* was found to decrease in the fish fed 5.0% β-glucan, relative to the control groups (Fig. 2). Carps receiving a diet supplemented with β-glucan showed the same levels of *Proteobacteria*, *Firmicutes,* and *Fusobacteria* [30]. When investigating the relative abundance of the bacteria at the genus level, higher concentrations of β-glucan appeared associated with an increased relative abundance of members of the genus *Aurantimicrobium*. Meanwhile, members of the genera *Carnobacterium* and *Deefgea* sp. were found at decreased relative abundance when the concentration of β-glucan increased in the diets. The genus *Aurantimicrobium* represents a new genus of aquatic bacteria, closely related to the genus *Alpinimonas*, and has only been described in 2015, following isolation from river water [53]. Since then, it has also been identified in the biofilter of marine recirculating aquaculture systems in the Netherlands [37]. There is little literature concerning *Aurantimicrobium* sp. as members of the fish microbiota, but it is likely due to the fact that the genus has only been described so recently. Moreover, the sequence of its 16S rRNA gene presents a relatively high level of similarity with that of *Alpinimonas psychrophila* (97.2%). However, more recently, there have been reports of this organisms within this intestinal microbiota of Nile tilapia (*Oreochromis niloticus*) and Crucian carp (*Carassius auratus*) [54, 55].

In addition, *Carnobacterium* was found to play a significant role in shaping the community in the control group, but its relative abundance decreased within the groups fed the highest doses of β-glucan. This observation is numerically confirmed by the relative read abundance in these groups, showing a reduction of this genus from 33.4% of the total community in the control group at week 6 to only 1.0% in the 5.0% β-glucan-treated group. *Carnobacterium* spp. are well-known members of the lactic acid bacteria (LAB) in the fish intestine. They are known to secrete bactericidal enzymes [56] and several members of this genus have shown potential as probiotic strains [57, 58]. Prebiotics act by promoting the growth of beneficial bacterial strains, so this could be an example of β-glucan working as intended. On the other hand, *Carnobacterium* sp. are also known to be associated with various diseases in fish, including rainbow trout [59, 60]. The genus *Deefgea*, belonging to the family *Neisseriaceae*, was also affected by the β-glucan treatment and also decreased in relative read abundance down to approximately 3.9% in the 5.0% β-glucan group, compared to 14.3% in the control group. *Deefgea*, and, in general, the family *Neisseriaceae*, are described as residents present in the gut of freshwater fish, but have not been associated with influencing the health of the host [61]. Two other LAB (*Lactobacillus* and *Bacillus*) were found in the gut microbiota and were also found to decrease at higher concentrations of the β-glucan treatment, which confirms previous studies in carp that also found reduced relative abundance of these organisms at higher concentrations of dietary β-glucan [30].

Conspicuously absent from our findings were members of the genus *Mycoplasma*. These bacteria were not detected in our samples, yet they have been repeatedly reported as important members of the intestinal microbiota of salmonids, including in Atlantic salmon (*Salmo salar*) where their abundance appeared correlated to weight gain in the fish, suggesting a beneficial effect of these bacteria [62]. *Mycoplasma* sp. have also been reported to dominate the intestinal microbiota of *O. mykiss* [50]. On the other hand, the importance of *Mycoplasma* sp. has not been universally reported. For example, screening of the intestinal microbiota of brown trout (*S. trutta*) did not detect the presence of operational taxonomic units associated with the phylum *Mycoplasmatota* (formerly known as *Tenericutes*) [63]. In *O. mykiss* investigation by Etyemez and Balcázar, sequencing amplificons of the V1–V3 region of the 16S rRNA gene, only identified *Mycoplasma* sp. at low abundance [64]. This is consistent with reports from Rimoldi et al. (that relied on the next generation sequencing of amplicons from the V3–V4 region of the 16S rRNA gene) that only found *Mycoplasma* sp. at very low levels [65] and that of Parshukov et al. [51] that reported that the presence of *Mycoplasma* sp. was low in most sampled *O. mykiss* and, in contradiction with the findings of Bozzi et al., associated with unhealthy fish. The reasons for this inconsistency are unknown but it is plausible that they are a combination of actual change in the microbiota of the fish, coupled with technical issues. The fact that several studies used the same primer sets and yet obtained different results suggest that primer bias is not the issue. On the other hand, Rimoldi et al. suggested that extraction bias linked with the peculiar structure of the *Mycoplasma* cell wall might play a role [65]. This is consistent with the fact that a change in the DNA extraction methods used by Rimoldi et al. between their 2018 and 2021 studies did correlate with different findings in terms of the abundance [50, 65]. This issue underscore what little we still know about the fish microbiota and the need for additional studies to better understand what is normal and to harmonise techniques, or at least better understand the effect of their intrinsic bias.

A statistical correlation of the β-glucan treatments and the microbial communities was established by multivariate data analysis and revealed a strong dependence on the β-glucan treatment (0.1%, 1.0%, and 5.0%), as illustrated by the clustering of the microbial communities, which became more clustered and less diverse at the highest concentrations of β-glucan, suggesting a strong effect of the β-glucan in driving the community when applied at that dose (Fig. 3). This is in accordance with previous findings in carp that confirm that addition of dietary β-glucan had a strong effect in the composition of the intestinal microbiota [30].

β-Glucan administration further resulted in significant up- or downregulation of multiple blood serum metabolites, most notably from the group of Phosphatidylcholines, which included 32 of 38 compounds that were significantly different, compared to the control group (*p* < 0.05) (Table S6). This group of phospholipids are major constituents of biological membranes and pulmonary surfactant known to play a vital role in membrane-mediating cell signalling [66]. Phosphatidylcholines can also play an immunostimulating role, assisting in the activation of several types of immune-cells, notably T-cells [67, 68], and have been linked to inflammatory and allergic responses [69]. Additional reported effects of Phosphatidylcholines cover oxidative damage, and Phosphatidylcholines have been proposed as a food supplement for slowing down aging-related processes [70]. Of the 32 Phosphatidylcholines that were significantly changed compared to the control, 31 were downregulated, and only Phosphatidylcholine with diacyl residue sum C24:0 was upregulated. The role of Phosphatidylcholines in aquatic organisms remains unexplored, and to the best of our knowledge, only a single study has shown that supplements of these to the diet can improve growth and reduce sensitivity toward osmotic stress in shrimps (*Penaeus vannamei*) [71]. Furthermore, an interesting observation is that a group of Lysophosphatidylcholines (4 compounds), a key factor in cardiovascular and neurodegenerative diseases, was found to be upregulated due to the administration of β-glucan. The gut microbiota has already been shown to influence both the intestinal lipidome of mice and that of the serum and liver, including concentrations of lysophosphatidylcholine [72–74]. Our present findings suggest that the lipidome of rainbow trout is similarly influenced by the intestinal microbiota and can therefore be affected by the addition of β-glucan in the diet.

The effect by Phosphatidylcholines, as well as the other compounds found to be significantly influenced by the addition of β-glucan to the fish (Sphingomyelin with acyl residue sum C18:1, Lysophosphatidylcholine with acyl residue C18:1, C18:2, C20:3, and C26:0, Hydroxysphingomyelin with acyl residue sum C14:1 and C22:2) remain to be further investigated.

Administration of the β-glucans had little measurable impact on the LC compounds, safe for alanine that was found to be upregulated in the fish subjected to the highest levels of β-glucan. While not statistically significant, with the exception of a single fish, nitrotyrosine was only detectable in the fish treated with the β-glucan. Nitrotyrosine is considered an indicator for the presence of nitrogen-free radical species [75] and so its elevated presence in the sera of the fish that had received the β-glucan could hint at increased oxidation. For example, this could be due to an increased release of superoxide compounds by macrophages and one could hypothesise that this is related to the increased production of phosphatidylcholines as these can have pro-inflammatory effects. This would be consistent with several studies that have reported an association between administration of β-glucan and the production of oxidative radicals by neutrophils in salmons [18] and in *O. mykiss* [76, 77]*.* However, because this change was not statistically significant, no interpretation can be made of this change. Inflammation would be expected to negatively impact the growth parameters of the fish, however, β-glucan are often associated with improved farming performances in fish, including in *O. mykiss* [22]*.* It is therefore possible that the cost of this inflammatory-like response is being compensated by other parameters; for example, a switch in the composition of the microbiota in a direction that is more favourable to the fish. These results show that addition of the β-glucans to the diet of the fish resulted in alteration of several of the serum’s metabolites and it is likely that these alterations might correlate to the improvement in fish farming performance and the immunostimulatory effects reported in association with β-glucans supplementation.

The disease did not result in severe clinical signs and very low mortality. Therefore, the resulting survival curves were not informative. Because both the infection dose and the exposure time were well within the ranges where *Y. ruckeri* can cause mortalities in *O. mykiss* [78, 79], it is likely that the bacterial isolate (*Y. ruckeri* O1 biotype 2 100415-1/4) was of low virulence. Indeed, other studies using the same isolate did not result in mortalities [80]. The only recorded mortalities occurred in the fish that had received the diet supplemented with the highest doses of β-glucan, although this difference was probably not meaningful. The prevalence of *Y. ruckeri* was also higher in these fish which could suggest that the β-glucan supplement resulted in a limited increase in the fish’ susceptibility to infection. This was unexpected as β-glucan are generally regarded as immunostimulatory [23, 81, 82], notably enhancing the resistance of rainbow trout against *Y. ruckeri* infections [83]. Under these circumstances, it seems more plausible that these non-significant results, only involving a small number of fish, are coincidental and that the susceptibility of the fish to *Y. ruckeri* infection was unchanged.

## Conclusions

In the present study, we investigated the effect of feed supplementation with β-glucans in rainbow trout. This supplementation led to significant changes in the composition of the intestinal microbiota, and in particular an increase in the relative abundance of sequence associated with the genus *Aurantimicrobium*. And a reduction in the relative abundance of the sequences associated with the genera *Carnobacterium* and *Deefgea* in the fish that had received the highest dose of β-glucan. These fish also displayed alterations in the serum levels of several metabolites, notably compounds related to phosphatidylcholines. Meanwhile, the treatment only had limited effect on the fish’ susceptibility to experimental infection with *Y. ruckeri.*

To our knowledge, this is the first study analysing the effect of β-glucan on the microbiota utilizing next generation sequencing and reporting on the role of the microbiota on the serum metabolome in rainbow trout.

## Supplementary Information

**Additional file 1: Table S1**. Allocation of the fish at the start of the experiment. **Table S2**. Analysis of the composition of the β-glucan used in this study. **Figure S3**. Principal component analysis showing the microbiota of the fish at week 6. **Figure S4.** Heatmap showing the 10 most abundant bacterial phyla in the intestine of the fish for the two control groups and the groups receiving the three different concentration of β-glucan. **Table S5.** Performance of the fish over the course of the experiment. **Figure S6.** Average weight of the fish per treatment over the course of the experiment. **Figure S7.** Average Fulton's condition factor (K) of the fish per treatment over the course of the experiment. **Table S8.** All metabolites measured in the serum of the fish for all investigated groups. **Table S9.** Metabolites measured at significantly different levels between treated fish and the control. Arrows indicate if the metabolite were upregulated (↑) or downregulated (↓) relative to the control without β-glucan treatment. Lack of significant change is indicated by “No”. **Figure S10**. Survival curves of the fish following 9 h bath exposure to 1.6 × 10^7^ CFU∙mL^−1^ of live *Y. ruckeri* O1 biotype 2 (100415-1/4).

## Data Availability

DNA sequences have been uploaded to the database of the European Nucleotide Archive under the project number PRJEB54041. Results from the metabolite analyses were uploaded to the UCSD Metabolomics Workbench under the reference numbers: 1805/DataTrackID3422 (for the lipids dataset) and 1805/DataTrackID3423 (for the amino acids dataset). The rest of datasets supporting the conclusions of this article are included within the article and its additional files or are available from the authors upon request.
