# Supplementary material for: Modulation of gut microbiota, blood metabolites, and disease resistance by dietary β-glucan in rainbow trout (Oncorhynchus mykiss)
Source: Anim Microbiome. 2022 Nov 20;4:58. doi: 10.1186/s42523-022-00209-5 (PMC9677660; doi:10.1186/s42523-022-00209-5)

Additional file 1

Additional file 1: Table S1. Allocation of the fish at the start of the experiment.

| **Name of the diet** | Infected | Number of tanks | Total number of fish |
| --- | --- | --- | --- |
| Control diet | N | 2 | 70 |
| Control diet | Y | 2 | 60 |
| 0.1% β-glucan | Y | 2 | 60 |
| 1.0% β-glucan | Y | 2 | 60 |
| 5.0% β-glucan | Y | 2 | 60 |

Additional file 1: Table S2. Analysis of the composition of the β-glucan used in this study.

| **Parameters** | **Unit** | **Value** |
| --- | --- | --- |
| Protein | % | 4.09 |
| Moisture | % | 7.95 |
| Ash | % | 2.53 |
| Fat | % | 4.10 |
| pH (2% sol.) |  | 6.40 |
| Glucan | % | 81.6 |
| Mannan | % | 1.5 |
| Ca | % | 0.08 |
| P | % | 0.09 |
| K | % | 0.07 |
| Na | % | 0.93 |
| Mg | % | 0.07 |
| Cu | mg/kg | 1.45 |
| Fe | mg/kg | 82.99 |
| Mn | mg/kg | 5.58 |
| Zn | mg/kg | 365.74 |
| Co | mg/kg | < 0.50 |
| Mo | mg/kg | < 0.50 |
| Ni | mg/kg | < 0.50 |
| Pb | mg/kg | < 0.50 |
| Cr | mg/kg | 0.6 |
| As | mg/kg | < 0.1 |
| Ba | mg/kg | 2.90 |
| Al | mg/kg | 57.81 |
| Cd | mg/kg | < 0.5 |
| S | % | 0.03 |
|  |  |  |
| Particle Size – Mean | µm | 37.7 |
| Particle Size >10um | % | 91.4 |
| Particle Size >20 um | % | 75.0 |
| Particle Size >50 um | % | 22.9 |
| Particle Size >100 um | % | 4.0 |
| Particle Size >200 um | % | 0.0 |
| Particle Size > 500 um | % | 0.0 |
| Particle Size >1000 um | % | 0.0 |

Additional file 1: Figure S3. Principal component analysis showing the microbiota of the fish at week 6.


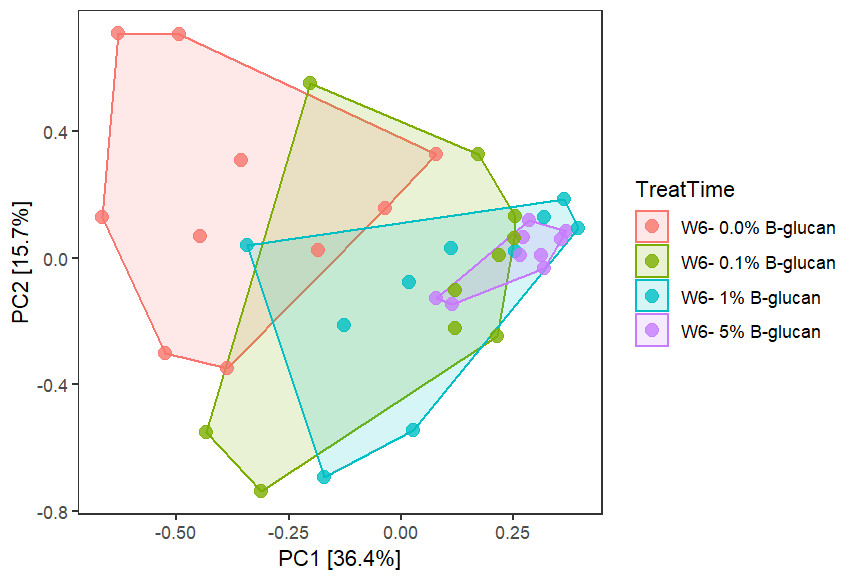


Additional file 1: Figure S4. Heatmap showing the 10 most abundant bacterial phyla in the intestine of the fish for the two control groups and the group receiving the three different concentration of β-glucan.


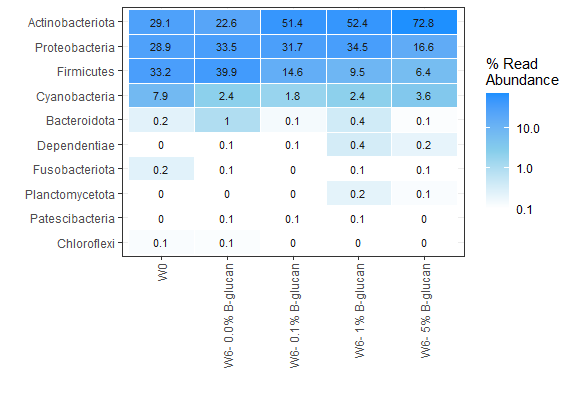


Additional file 1: Table S5. Performance of the fish over the course of the experiment

| **Date** | **Days post-infection** | **Treatment** | **Mass_total_ (g)** | **L_standard_ (cm)** | **L_total_ (cm)** | **Fulton's condition factor (*K*)** | **Mean *K*** | **Mean mass_total_ (g)** |
| --- | --- | --- | --- | --- | --- | --- | --- | --- |
| Week 0 | - | 0% β-glucan | 19.3 | 11.1 | 12.3 | 1.41 |  |  |
| Week 0 | - | 0% β-glucan | 14.5 | 9.7 | 11.0 | 1.59 |  |  |
| Week 0 | - | 0% β-glucan | 13.7 | 9.3 | 11.0 | 1.70 |  |  |
| Week 0 | - | 0% β-glucan | 15.8 | 10.3 | 11.1 | 1.45 |  |  |
| Week 0 | - | 0% β-glucan | 13.2 | 9.6 | 10.7 | 1.49 |  |  |
| Week 0 | - | 0% β-glucan | 19.3 | 11.1 | 12.2 | 1.41 |  |  |
| Week 0 | - | 0% β-glucan | 9.1 | 8.4 | 9.3 | 1.54 |  |  |
| Week 0 | - | 0% β-glucan | 20.6 | 11.1 | 12.4 | 1.51 |  |  |
| Week 0 | - | 0% β-glucan | 15.1 | 9.8 | 10.9 | 1.60 |  |  |
| Week 0 | - | 0% β-glucan | 14.5 | 9.8 | 10.9 | 1.54 | **1.52** | **15.51** |
|  |  |  |  |  |  |  |  |  |
| Week 3 | - | 0% β-glucan | 19.5 | - | - | - |  |  |
| Week 3 | - | 0% β-glucan | 19.0 | - | - | - |  |  |
| Week 3 | - | 0% β-glucan | 21.5 | - | - | - |  |  |
| Week 3 | - | 0% β-glucan | 22.0 | - | - | - |  |  |
| Week 3 | - | 0% β-glucan | 17.0 | - | - | - |  |  |
| Week 3 | - | 0% β-glucan | 20.5 | - | - | - |  |  |
| Week 3 | - | 0% β-glucan | 15.5 | - | - | - |  |  |
| Week 3 | - | 0% β-glucan | 12.5 | - | - | - |  |  |
| Week 3 | - | 0% β-glucan | 25.5 | - | - | - |  |  |
| Week 3 | - | 0% β-glucan | 12.5 | - | - | - |  | **18.6** |
| Week 3 | - | 0.1% β-glucan | 15.0 | - | - | - |  |  |
| Week 3 | - | 0.1% β-glucan | 21.0 | - | - | - |  |  |
| Week 3 | - | 0.1% β-glucan | 20.5 | - | - | - |  |  |
| Week 3 | - | 0.1% β-glucan | 19.5 | - | - | - |  |  |
| Week 3 | - | 0.1% β-glucan | 13.5 | - | - | - |  |  |
| Week 3 | - | 0.1% β-glucan | - | - | - | - |  |  |
| Week 3 | - | 0.1% β-glucan | 16.5 | - | - | - |  |  |
| Week 3 | - | 0.1% β-glucan | 19.5 | - | - | - |  |  |
| Week 3 | - | 0.1% β-glucan | 33.0 | - | - | - |  |  |
| Week 3 | - | 0.1% β-glucan | 17.5 | - | - | - |  | **19.6** |
| Week 3 | - | 1.0% β-glucan | 21.0 | - | - | - |  |  |
| Week 3 | - | 1.0% β-glucan | 22.5 | - | - | - |  |  |
| Week 3 | - | 1.0% β-glucan | 15.0 | - | - | - |  |  |
| Week 3 | - | 1.0% β-glucan | - | - | - | - |  |  |
| Week 3 | - | 1.0% β-glucan | - | - | - | - |  |  |
| Week 3 | - | 1.0% β-glucan | 17.5 | - | - | - |  |  |
| Week 3 | - | 1.0% β-glucan | - | - | - | - |  |  |
| Week 3 | - | 1.0% β-glucan | 15.5 | - | - | - |  |  |
| Week 3 | - | 1.0% β-glucan | 27.5 | - | - | - |  |  |
| Week 3 | - | 1.0% β-glucan | 20.0 | - | - | - |  | **19.9** |
| Week 3 | - | 5.0% β-glucan | 15.0 | - | - | - |  |  |
| Week 3 | - | 5.0% β-glucan | 20.0 | - | - | - |  |  |
| Week 3 | - | 5.0% β-glucan | 18.0 | - | - | - |  |  |
| Week 3 | - | 5.0% β-glucan | 22.0 | - | - | - |  |  |
| Week 3 | - | 5.0% β-glucan | 13.0 | - | - | - |  |  |
| Week 3 | - | 5.0% β-glucan | 29.0 | - | - | - |  |  |
| Week 3 | - | 5.0% β-glucan | 14.0 | - | - | - |  |  |
| Week 3 | - | 5.0% β-glucan | 12.5 | - | - | - |  |  |
| Week 3 | - | 5.0% β-glucan | 20.5 | - | - | - |  |  |
| Week 3 | - | 5.0% β-glucan | 19.5 | - | - | - |  | **18.4** |
|  |  |  |  |  |  |  |  |  |
| Week 6 | 0 | 0% β-glucan | 27.0 | 11.8 | 13.1 | 1.64 |  |  |
| Week 6 | 0 | 0% β-glucan | 24.5 | 12.4 | 13.1 | 1.28 |  |  |
| Week 6 | 0 | 0% β-glucan | 17.5 | 10.7 | 12.0 | 1.43 |  |  |
| Week 6 | 0 | 0% β-glucan | 16.5 | 10.3 | 11.4 | 1.51 |  |  |
| Week 6 | 0 | 0% β-glucan | 24.5 | 12.2 | 13.0 | 1.35 |  |  |
| Week 6 | 0 | 0% β-glucan | 32.0 | 13.3 | 14.6 | 1.36 |  |  |
| Week 6 | 0 | 0% β-glucan | 18.0 | 10.6 | 11.9 | 1.51 |  |  |
| Week 6 | 0 | 0% β-glucan | 21.0 | 11.2 | 12.6 | 1.49 |  |  |
| Week 6 | 0 | 0% β-glucan | 28.5 | 12.9 | 14.2 | 1.33 |  |  |
| Week 6 | 0 | 0% β-glucan | 14.5 | 9.8 | 10.9 | 1.54 | **1.45** | **22.4** |
| Week 6 | 0 | 0.1% β-glucan | 18.0 | 10.7 | 11.9 | 1.47 |  |  |
| Week 6 | 0 | 0.1% β-glucan | 23.5 | 11.4 | 12.5 | 1.59 |  |  |
| Week 6 | 0 | 0.1% β-glucan | 19.0 | 10.9 | 12.1 | 1.47 |  |  |
| Week 6 | 0 | 0.1% β-glucan | 26.0 | 12.4 | 13.6 | 1.36 |  |  |
| Week 6 | 0 | 0.1% β-glucan | 20.0 | 11.0 | 12.1 | 1.50 |  |  |
| Week 6 | 0 | 0.1% β-glucan | 14.5 | 10.0 | 11.2 | 1.45 |  |  |
| Week 6 | 0 | 0.1% β-glucan | 14.5 | 9.5 | 10.6 | 1.69 |  |  |
| Week 6 | 0 | 0.1% β-glucan | 23.0 | 12.0 | 13.3 | 1.33 |  |  |
| Week 6 | 0 | 0.1% β-glucan | 30.5 | 12.5 | 13.7 | 1.56 |  |  |
| Week 6 | 0 | 0.1% β-glucan | 15.0 | 10.0 | 11.2 | 1.50 | **1.49** | **20.4** |
| Week 6 | 0 | 1.0% β-glucan | 17.0 | 10.5 | 11.6 | 1.47 |  |  |
| Week 6 | 0 | 1.0% β-glucan | 16.0 | 9.5 | 10.7 | 1.87 |  |  |
| Week 6 | 0 | 1.0% β-glucan | 22.0 | 11.4 | 12.8 | 1.48 |  |  |
| Week 6 | 0 | 1.0% β-glucan | 17.5 | 10.5 | 11.5 | 1.51 |  |  |
| Week 6 | 0 | 1.0% β-glucan | 22.5 | 11.5 | 12.6 | 1.48 |  |  |
| Week 6 | 0 | 1.0% β-glucan | 30.5 | 12.8 | 14.2 | 1.45 |  |  |
| Week 6 | 0 | 1.0% β-glucan | 25.0 | 11.8 | 13.4 | 1.52 |  |  |
| Week 6 | 0 | 1.0% β-glucan | 31.5 | 13.1 | 14.5 | 1.40 |  |  |
| Week 6 | 0 | 1.0% β-glucan | 22.5 | 11.8 | 13.1 | 1.37 |  |  |
| Week 6 | 0 | 1.0% β-glucan | 13.0 | 9.4 | 10.6 | 1.57 | **1.51** | **21.8** |
| Week 6 | 0 | 5.0% β-glucan | 30.5 | 13.2 | 14.5 | 1.33 |  |  |
| Week 6 | 0 | 5.0% β-glucan | 29.0 | 13.0 | 14.4 | 1.32 |  |  |
| Week 6 | 0 | 5.0% β-glucan | 19.0 | 10.7 | 11.9 | 1.55 |  |  |
| Week 6 | 0 | 5.0% β-glucan | 17.0 | 10.4 | 11.6 | 1.51 |  |  |
| Week 6 | 0 | 5.0% β-glucan | 30.5 | 13.0 | 14.2 | 1.39 |  |  |
| Week 6 | 0 | 5.0% β-glucan | 23.5 | 11.7 | 12.7 | 1.47 |  |  |
| Week 6 | 0 | 5.0% β-glucan | 13.5 | 9.6 | 10.8 | 1.53 |  |  |
| Week 6 | 0 | 5.0% β-glucan | 17.5 | 10.7 | 11.7 | 1.43 |  |  |
| Week 6 | 0 | 5.0% β-glucan | 20.5 | 11.3 | 12.6 | 1.42 |  |  |
| Week 6 | 0 | 5.0% β-glucan | 13.0 | 9.9 | 10.9 | 1.34 | **1.43** | **21.4** |
|  |  |  |  |  |  |  |  |  |
| Week 6 | 0 | 0% β-glucan (uninfected control) | 30.5 | 13.3 | 14.8 | 1.30 |  |  |
| Week 6 | 0 | 0% β-glucan (uninfected control) | 22.5 | 11.3 | 12.1 | 1.56 |  |  |
| Week 6 | 0 | 0% β-glucan (uninfected control) | 20.5 | 11.4 | 12.7 | 1.38 |  |  |
| Week 6 | 0 | 0% β-glucan (uninfected control) | 32.0 | 13.0 | 14.4 | 1.46 |  |  |
| Week 6 | 0 | 0% β-glucan (uninfected control) | 17.5 | 10.0 | 11.7 | 1.75 |  |  |
| Week 6 | 0 | 0% β-glucan (uninfected control) | 22.0 | 11.0 | 12.4 | 1.65 |  |  |
| Week 6 | 0 | 0% β-glucan (uninfected control) | 25.5 | 12.0 | 13.4 | 1.48 |  |  |
| Week 6 | 0 | 0% β-glucan (uninfected control) | 24.5 | 12.0 | 13.4 | 1.42 |  |  |
| Week 6 | 0 | 0% β-glucan (uninfected control) | 23.5 | 11.4 | 12.7 | 1.59 |  |  |
| Week 6 | 0 | 0% β-glucan (uninfected control) | 34.5 | 12.8 | 14.1 | 1.65 | **1.52** | **25.3** |
| Week 6 | 0 | 0% β-glucan | 24.0 | 11.9 | 13.2 | 1.42 |  |  |
| Week 6 | 0 | 0% β-glucan | 18.5 | 11.2 | 12.5 | 1.32 |  |  |
| Week 6 | 0 | 0% β-glucan | 18.0 | - | - | - |  |  |
| Week 6 | 0 | 0% β-glucan | 26.5 | 12.4 | 13.6 | 1.39 |  |  |
| Week 6 | 0 | 0% β-glucan | 26.5 | 12.3 | 13.5 | 1.42 |  |  |
| Week 6 | 0 | 0% β-glucan | 17.0 | 10.7 | 11.1 | 1.39 |  |  |
| Week 6 | 0 | 0% β-glucan | 27.5 | 13.0 | 14.4 | 1.25 |  |  |
| Week 6 | 0 | 0% β-glucan | 25.5 | 11.8 | 13.1 | 1.55 |  |  |
| Week 6 | 0 | 0% β-glucan | 14.0 | 9.5 | 10.5 | 1.63 |  |  |
| Week 6 | 0 | 0% β-glucan | 13.5 | 9.7 | 10.8 | 1.48 | **1.43** | **21.1** |
| Week 6 | 0 | 0.1% β-glucan | 38.5 | 13.5 | 14.9 | 1.56 |  |  |
| Week 6 | 0 | 0.1% β-glucan | 22.0 | 11.5 | 12.8 | 1.45 |  |  |
| Week 6 | 0 | 0.1% β-glucan | 23.5 | 11.7 | 13.1 | 1.47 |  |  |
| Week 6 | 0 | 0.1% β-glucan | 16.0 | 10.0 | 11.2 | 1.60 |  |  |
| Week 6 | 0 | 0.1% β-glucan | 14.0 | 9.9 | 10.9 | 1.44 |  |  |
| Week 6 | 0 | 0.1% β-glucan | 21.5 | 11.7 | 12.9 | 1.34 |  |  |
| Week 6 | 0 | 0.1% β-glucan | 22.5 | 11.1 | 12.3 | 1.65 |  |  |
| Week 6 | 0 | 0.1% β-glucan | 36.0 | 13.6 | 15.1 | 1.43 |  |  |
| Week 6 | 0 | 0.1% β-glucan | 22.0 | 11.9 | 13.2 | 1.31 |  |  |
| Week 6 | 0 | 0.1% β-glucan | 30.5 | 12.7 | 14.1 | 1.49 | **1.47** | **24.7** |
| Week 6 | 0 | 1.0% β-glucan | 15.5 | 10.2 | 11.5 | 1.46 |  |  |
| Week 6 | 0 | 1.0% β-glucan | 26.0 | 11.9 | 13.1 | 1.54 |  |  |
| Week 6 | 0 | 1.0% β-glucan | 36.5 | 14.0 | 15.4 | 1.33 |  |  |
| Week 6 | 0 | 1.0% β-glucan | 18.0 | 11.0 | 12.2 | 1.35 |  |  |
| Week 6 | 0 | 1.0% β-glucan | 24.0 | 11.5 | 12.9 | 1.58 |  |  |
| Week 6 | 0 | 1.0% β-glucan | 25.0 | 12.2 | 13.1 | 1.38 |  |  |
| Week 6 | 0 | 1.0% β-glucan | 24.5 | 12.0 | 13.2 | 1.42 |  |  |
| Week 6 | 0 | 1.0% β-glucan | 24.5 | 12.1 | 13.4 | 1.38 |  |  |
| Week 6 | 0 | 1.0% β-glucan | 15.0 | 10.1 | 11.3 | 1.46 |  |  |
| Week 6 | 0 | 1.0% β-glucan | 21.0 | 11.2 | 12.4 | 1.49 | **1.44** | **23.0** |
| Week 6 | 0 | 5.0% β-glucan | 15.0 | 9.8 | 11.0 | 1.59 |  |  |
| Week 6 | 0 | 5.0% β-glucan | 17.5 | 10.6 | 11.9 | 1.47 |  |  |
| Week 6 | 0 | 5.0% β-glucan | 14.0 | 10.3 | 11.6 | 1.28 |  |  |
| Week 6 | 0 | 5.0% β-glucan | 28.0 | 13.0 | 14.2 | 1.27 |  |  |
| Week 6 | 0 | 5.0% β-glucan | 22.0 | 11.9 | 13.1 | 1.31 |  |  |
| Week 6 | 0 | 5.0% β-glucan | 16.0 | 10.4 | 11.5 | 1.42 |  |  |
| Week 6 | 0 | 5.0% β-glucan | 19.5 | 11.0 | 12.2 | 1.47 |  |  |
| Week 6 | 0 | 5.0% β-glucan | 23.0 | 11.8 | 13.0 | 1.40 |  |  |
| Week 6 | 0 | 5.0% β-glucan | 27.5 | 12.5 | 13.8 | 1.41 |  |  |
| Week 6 | 0 | 5.0% β-glucan | 38.5 | 13.6 | 15.0 | 1.53 | **1.42** | **22.1** |
| Week 6 |  |  |  |  |  |  |  |  |
| Week 6 | 0 | 0% β-glucan (uninfected control) | 26.5 | 12.2 | 13.5 | 1.46 |  |  |
| Week 6 | 0 | 0% β-glucan (uninfected control) | 31.5 | 13.0 | 14.2 | 1.43 |  |  |
| Week 6 | 0 | 0% β-glucan (uninfected control) | 21.0 | 11.2 | 12.6 | 1.49 |  |  |
| Week 6 | 0 | 0% β-glucan (uninfected control) | 29.5 | 12.6 | 14.1 | 1.47 |  |  |
| Week 6 | 0 | 0% β-glucan (uninfected control) | 21.0 | 11.6 | 13.0 | 1.35 |  |  |
| Week 6 | 0 | 0% β-glucan (uninfected control) | 23.0 | 11.6 | 12.8 | 1.47 |  |  |
| Week 6 | 0 | 0% β-glucan (uninfected control) | 17.0 | 10.2 | 11.3 | 1.60 |  |  |
| Week 6 | 0 | 0% β-glucan (uninfected control) | 22.0 | 11.1 | 12.5 | 1.61 |  |  |
| Week 6 | 0 | 0% β-glucan (uninfected control) | 26.0 | 12.3 | 13.6 | 1.40 |  |  |
| Week 6 | 0 | 0% β-glucan (uninfected control) | 37.0 | 13.9 | 15.4 | 1.38 | **1.47** | **25.5** |
| Week 6 | 0 | 0% β-glucan | 17.5 | 10.5 | 11.7 | 1.51 |  |  |
| Week 6 | 0 | 0% β-glucan | 22.0 | 11.3 | 12.3 | 1.52 |  |  |
| Week 6 | 0 | 0% β-glucan | 33.0 | 13.3 | 14.7 | 1.40 |  |  |
| Week 6 | 0 | 0% β-glucan | 27.0 | 12.5 | 14.0 | 1.38 |  |  |
| Week 6 | 0 | 0% β-glucan | 30.5 | - | - | - |  |  |
| Week 6 | 0 | 0% β-glucan | 19.0 | 10.8 | 11.9 | 1.51 |  |  |
| Week 6 | 0 | 0% β-glucan | 36.0 | 14.1 | 15.6 | 1.28 |  |  |
| Week 6 | 0 | 0% β-glucan | 21.5 | 11.4 | 12.7 | 1.45 |  |  |
| Week 6 | 0 | 0% β-glucan | 23.5 | 12.3 | 13.7 | 1.26 |  |  |
| Week 6 | 0 | 0% β-glucan | 19.0 | 10.8 | 12.0 | 1.51 | **1.43** | **24.9** |
| Week 6 | 0 | 0.1% β-glucan | 24.0 | 11.7 | 13.0 | 1.50 |  |  |
| Week 6 | 0 | 0.1% β-glucan | 29.5 | 12.5 | 13.7 | 1.51 |  |  |
| Week 6 | 0 | 0.1% β-glucan | 23.0 | 11.7 | 13.0 | 1.44 |  |  |
| Week 6 | 0 | 0.1% β-glucan | 20.5 | 11.5 | 12.8 | 1.35 |  |  |
| Week 6 | 0 | 0.1% β-glucan | 20.5 | 10.9 | 11.9 | 1.58 |  |  |
| Week 6 | 0 | 0.1% β-glucan | 31.0 | 12.8 | 13.8 | 1.48 |  |  |
| Week 6 | 0 | 0.1% β-glucan | 13.5 | 9.5 | 10.5 | 1.57 |  |  |
| Week 6 | 0 | 0.1% β-glucan | 16.0 | 10.3 | 11.5 | 1.46 |  |  |
| Week 6 | 0 | 0.1% β-glucan | 21.5 | 11.5 | 13.0 | 1.41 |  |  |
| Week 6 | 0 | 0.1% β-glucan | 15.0 | 10.4 | 11.5 | 1.33 | **1.46** | **21.5** |
| Week 6 | 0 | 1.0% β-glucan | 18.0 | 10.4 | 11.8 | 1.60 |  |  |
| Week 6 | 0 | 1.0% β-glucan | 29.0 | 12.5 | 13.8 | 1.48 |  |  |
| Week 6 | 0 | 1.0% β-glucan | 21.5 | 10.5 | 11.8 | 1.86 |  |  |
| Week 6 | 0 | 1.0% β-glucan | 21.0 | 11.2 | 12.5 | 1.49 |  |  |
| Week 6 | 0 | 1.0% β-glucan | 25.0 | 12.3 | 13.6 | 1.34 |  |  |
| Week 6 | 0 | 1.0% β-glucan | 25.0 | 12.2 | 13.4 | 1.38 |  |  |
| Week 6 | 0 | 1.0% β-glucan | 15.5 | 10.1 | 11.2 | 1.50 |  |  |
| Week 6 | 0 | 1.0% β-glucan | 25.0 | 12.4 | 13.8 | 1.31 |  |  |
| Week 6 | 0 | 1.0% β-glucan | 21.0 | 11.4 | 12.7 | 1.42 |  |  |
| Week 6 | 0 | 1.0% β-glucan | 24.5 | 12.3 | 13.2 | 1.32 | **1.47** | **22.6** |
| Week 6 | 0 | 5.0% β-glucan | 37.0 | 13.7 | 15.0 | 1.44 |  |  |
| Week 6 | 0 | 5.0% β-glucan | 21.5 | 11.3 | 12.5 | 1.49 |  |  |
| Week 6 | 0 | 5.0% β-glucan | 22.5 | 11.8 | 13.1 | 1.37 |  |  |
| Week 6 | 0 | 5.0% β-glucan | 13.5 | 10.0 | 11.2 | 1.35 |  |  |
| Week 6 | 0 | 5.0% β-glucan | 17.0 | 10.4 | 11.7 | 1.51 |  |  |
| Week 6 | 0 | 5.0% β-glucan | 16.5 | 10.2 | 11.3 | 1.55 |  |  |
| Week 6 | 0 | 5.0% β-glucan | 25.0 | 11.7 | 13.0 | 1.56 |  |  |
| Week 6 | 0 | 5.0% β-glucan | 26.5 | 12.2 | 13.4 | 1.46 |  |  |
| Week 6 | 0 | 5.0% β-glucan | 33.5 | 12.9 | 14.3 | 1.56 |  |  |
| Week 6 | 0 | 5.0% β-glucan | 15.0 | 9.7 | 10.9 | 1.64 | **1.49** | **22.8** |
| Week 6 |  |  |  |  |  |  |  |  |
| Week 6 | 0 | 0% β-glucan (uninfected control) | 28.5 | 12.2 | 13.5 | 1.57 |  |  |
| Week 6 | 0 | 0% β-glucan (uninfected control) | 16.5 | 10.4 | 11.6 | 1.47 |  |  |
| Week 6 | 0 | 0% β-glucan (uninfected control) | 25.5 | 11.8 | 13.3 | 1.55 |  |  |
| Week 6 | 0 | 0% β-glucan (uninfected control) | 20.0 | 11.0 | 12.3 | 1.50 |  |  |
| Week 6 | 0 | 0% β-glucan (uninfected control) | 24.0 | 11.7 | 12.8 | 1.50 |  |  |
| Week 6 | 0 | 0% β-glucan (uninfected control) | 17.5 | 10.5 | 11.7 | 1.51 |  |  |
| Week 6 | 0 | 0% β-glucan (uninfected control) | 16.0 | 9.7 | 11.0 | 1.75 |  |  |
| Week 6 | 0 | 0% β-glucan (uninfected control) | 26.0 | 12.1 | 13.5 | 1.47 |  |  |
| Week 6 | 0 | 0% β-glucan (uninfected control) | 20.0 | 10.9 | 12.1 | 1.54 |  |  |
| Week 6 | 0 | 0% β-glucan (uninfected control) | 20.0 | 10.4 | 11.5 | 1.78 | **1.56** | **21.4** |
| Week 6 | 0 | 0% β-glucan | 27.5 | 12.5 | 13.7 | 1.41 |  |  |
| Week 6 | 0 | 0% β-glucan | 23.0 | 11.9 | 13.4 | 1.36 |  |  |
| Week 6 | 0 | 0% β-glucan | 22.5 | 11.7 | 13.0 | 1.40 |  |  |
| Week 6 | 0 | 0% β-glucan | 32.0 | 13.0 | 14.3 | 1.46 |  |  |
| Week 6 | 0 | 0% β-glucan | 23.0 | 11.9 | 13.2 | 1.36 |  |  |
| Week 6 | 0 | 0% β-glucan | 35.0 | 13.5 | 14.8 | 1.42 |  |  |
| Week 6 | 0 | 0% β-glucan | 26.5 | 12.4 | 13.6 | 1.39 |  |  |
| Week 6 | 0 | 0% β-glucan | 28.0 | 12.5 | 13.7 | 1.43 |  |  |
| Week 6 | 0 | 0% β-glucan | 21.5 | 11.5 | 12.9 | 1.41 |  |  |
| Week 6 | 0 | 0% β-glucan | 26.5 | 12.5 | 13.8 | 1.36 | **1.40** | **26.6** |
| Week 6 | 0 | 0.1% β-glucan | 29.5 | 12.8 | 14.1 | 1.41 |  |  |
| Week 6 | 0 | 0.1% β-glucan | 18.0 | 10.6 | 11.7 | 1.51 |  |  |
| Week 6 | 0 | 0.1% β-glucan | 25.0 | 12.1 | 13.3 | 1.41 |  |  |
| Week 6 | 0 | 0.1% β-glucan | 18.0 | 10.3 | 11.5 | 1.65 |  |  |
| Week 6 | 0 | 0.1% β-glucan | 17.0 | 10.2 | 11.8 | 1.60 |  |  |
| Week 6 | 0 | 0.1% β-glucan | 16.0 | 10.0 | 11.3 | 1.60 |  |  |
| Week 6 | 0 | 0.1% β-glucan | 30.0 | 12.8 | 14.1 | 1.43 |  |  |
| Week 6 | 0 | 0.1% β-glucan | 21.0 | 11.1 | 12.1 | 1.54 |  |  |
| Week 6 | 0 | 0.1% β-glucan | 28.5 | 12.5 | 13.9 | 1.46 |  |  |
| Week 6 | 0 | 0.1% β-glucan | 25.5 | 12.5 | 13.8 | 1.31 | **1.49** | **22.9** |
| Week 6 | 0 | 1.0% β-glucan | 24.5 | 11.9 | 13.0 | 1.45 |  |  |
| Week 6 | 0 | 1.0% β-glucan | 16.0 | 10.0 | 11.2 | 1.60 |  |  |
| Week 6 | 0 | 1.0% β-glucan | 22.5 | 11.7 | 12.9 | 1.40 |  |  |
| Week 6 | 0 | 1.0% β-glucan | 13.5 | 9.8 | 10.9 | 1.43 |  |  |
| Week 6 | 0 | 1.0% β-glucan | 22.5 | 11.5 | 12.7 | 1.48 |  |  |
| Week 6 | 0 | 1.0% β-glucan | 34.5 | 13.6 | 15.0 | 1.37 |  |  |
| Week 6 | 0 | 1.0% β-glucan | 15.5 | 10.2 | 11.4 | 1.46 |  |  |
| Week 6 | 0 | 1.0% β-glucan | 32.5 | 13.6 | 14.8 | 1.29 |  |  |
| Week 6 | 0 | 1.0% β-glucan | 26.0 | 12.2 | 13.5 | 1.43 |  |  |
| Week 6 | 0 | 1.0% β-glucan | 32.0 | 12.9 | 14.2 | 1.49 | **1.44** | **24.0** |
| Week 6 | 0 | 5.0% β-glucan | 17.0 | 10.6 | 11.8 | 1.43 |  |  |
| Week 6 | 0 | 5.0% β-glucan | 24.0 | 11.7 | 13.0 | 1.50 |  |  |
| Week 6 | 0 | 5.0% β-glucan | 24.0 | 11.6 | 12.9 | 1.54 |  |  |
| Week 6 | 0 | 5.0% β-glucan | 15.5 | 10.1 | 11.3 | 1.50 |  |  |
| Week 6 | 0 | 5.0% β-glucan | 25.5 | 12.0 | 13.1 | 1.48 |  |  |
| Week 6 | 0 | 5.0% β-glucan | 24.0 | 12.0 | 13.4 | 1.39 |  |  |
| Week 6 | 0 | 5.0% β-glucan | 32.5 | 13.3 | 14.5 | 1.38 |  |  |
| Week 6 | 0 | 5.0% β-glucan | 28.5 | 12.2 | 13.4 | 1.57 |  |  |
| Week 6 | 0 | 5.0% β-glucan | 18.5 | 10.8 | 12.1 | 1.47 |  |  |
| Week 6 | 0 | 5.0% β-glucan | 32.0 | 13.0 | 14.7 | 1.46 | **1.47** | **24.2** |
|  |  |  |  |  |  |  |  |  |
| Week 8 | 14 | 0% β-glucan (uninfected control) | 18.5 | 10.8 | 12.1 | 1.47 |  |  |
| Week 8 | 14 | 0% β-glucan (uninfected control) | 24.5 | 11.9 | 13.0 | 1.45 |  |  |
| Week 8 | 14 | 0% β-glucan (uninfected control) | 18.0 | 10.8 | 11.9 | 1.43 |  |  |
| Week 8 | 14 | 0% β-glucan (uninfected control) | 32.5 | 13.1 | 14.5 | 1.45 |  |  |
| Week 8 | 14 | 0% β-glucan (uninfected control) | 17.5 | 10.0 | 11.3 | 1.75 |  |  |
| Week 8 | 14 | 0% β-glucan (uninfected control) | 30.5 | 12.5 | 13.7 | 1.56 |  |  |
| Week 8 | 14 | 0% β-glucan (uninfected control) | 25.0 | 11.8 | 13.2 | 1.52 |  |  |
| Week 8 | 14 | 0% β-glucan (uninfected control) | 17.5 | 10.1 | 11.2 | 1.70 |  |  |
| Week 8 | 14 | 0% β-glucan (uninfected control) | 23.5 | 11.2 | 12.6 | 1.67 |  |  |
| Week 8 | 14 | 0% β-glucan (uninfected control) | 22.5 | 11.3 | 12.5 | 1.56 | **1.56** | **23.0** |
| Week 8 | 14 | 0% β-glucan | 30.5 | 12.8 | 14.0 | 1.45 |  |  |
| Week 8 | 14 | 0% β-glucan | 31.5 | 12.9 | 14.2 | 1.47 |  |  |
| Week 8 | 14 | 0% β-glucan | 42.5 | 14.6 | 15.8 | 1.37 |  |  |
| Week 8 | 14 | 0% β-glucan | 22.5 | 11.7 | 12.7 | 1.40 |  |  |
| Week 8 | 14 | 0% β-glucan | 32.5 | 13.3 | 14.7 | 1.38 |  |  |
| Week 8 | 14 | 0% β-glucan | 30.0 | 12.6 | 13.8 | 1.50 |  |  |
| Week 8 | 14 | 0% β-glucan | 37.5 | 13.7 | 14.9 | 1.46 |  |  |
| Week 8 | 14 | 0% β-glucan | 26.0 | 12.6 | 14.0 | 1.30 |  |  |
| Week 8 | 14 | 0% β-glucan | 27.0 | 12.4 | 13.6 | 1.42 |  |  |
| Week 8 | 14 | 0% β-glucan | 19.5 | 11.1 | 12.2 | 1.43 |  |  |
| Week 8 | 14 | 0% β-glucan | 36.5 | 13.2 | 14.5 | 1.59 |  |  |
| Week 8 | 14 | 0% β-glucan | 29.5 | 13.0 | 14.5 | 1.34 |  |  |
| Week 8 | 14 | 0% β-glucan | 18.5 | 10.4 | 11.6 | 1.64 |  |  |
| Week 8 | 14 | 0% β-glucan | 16.5 | 10.2 | 11.4 | 1.55 |  |  |
| Week 8 | 14 | 0% β-glucan | 29.0 | 12.7 | 14.0 | 1.42 |  |  |
| Week 8 | 14 | 0% β-glucan | 19.0 | 10.8 | 12.1 | 1.51 |  |  |
| Week 8 | 14 | 0% β-glucan | 19.5 | 11.0 | 12.2 | 1.47 |  |  |
| Week 8 | 14 | 0% β-glucan | 28.0 | 12.5 | 13.6 | 1.43 |  |  |
| Week 8 | 14 | 0% β-glucan | 29.5 | 12.9 | 14.2 | 1.37 |  |  |
| Week 8 | 14 | 0% β-glucan | 30.5 | 12.7 | 14.1 | 1.49 | **1.45** | **27.8** |
| Week 8 | 14 | 0.1% β-glucan | 49.0 | 14.5 | 16.0 | 1.61 |  |  |
| Week 8 | 14 | 0.1% β-glucan | 23.0 | 11.7 | 12.8 | 1.44 |  |  |
| Week 8 | 14 | 0.1% β-glucan | 26.5 | 12.1 | 13.4 | 1.50 |  |  |
| Week 8 | 14 | 0.1% β-glucan | 18.5 | 11.0 | 12.2 | 1.39 |  |  |
| Week 8 | 14 | 0.1% β-glucan | 18.5 | 10.5 | 11.8 | 1.60 |  |  |
| Week 8 | 14 | 0.1% β-glucan | 32.5 | 13.3 | 14.3 | 1.38 |  |  |
| Week 8 | 14 | 0.1% β-glucan | 40.5 | 14.1 | 15.4 | 1.44 |  |  |
| Week 8 | 14 | 0.1% β-glucan | 22.0 | 11.7 | 12.9 | 1.37 |  |  |
| Week 8 | 14 | 0.1% β-glucan | 17.5 | 10.4 | 11.6 | 1.56 |  |  |
| Week 8 | 14 | 0.1% β-glucan | 14.0 | 10.0 | 11.2 | 1.40 |  |  |
| Week 8 | 14 | 0.1% β-glucan | 22.5 | 11.7 | 12.9 | 1.40 |  |  |
| Week 8 | 14 | 0.1% β-glucan | 17.0 | 10.5 | 11.8 | 1.47 |  |  |
| Week 8 | 14 | 0.1% β-glucan | 30.0 | 12.5 | 14.0 | 1.54 |  |  |
| Week 8 | 14 | 0.1% β-glucan | 29.0 | 12.8 | 13.9 | 1.38 |  |  |
| Week 8 | 14 | 0.1% β-glucan | 21.5 | 11.5 | 12.7 | 1.41 |  |  |
| Week 8 | 14 | 0.1% β-glucan | 26.0 | 12.1 | 13.3 | 1.47 |  |  |
| Week 8 | 14 | 0.1% β-glucan | 25.5 | 12.2 | 13.5 | 1.40 |  |  |
| Week 8 | 14 | 0.1% β-glucan | 39.5 | 13.4 | 14.7 | 1.64 |  |  |
| Week 8 | 14 | 0.1% β-glucan | 14.5 | 9.9 | 11.0 | 1.49 |  |  |
| Week 8 | 14 | 0.1% β-glucan | 17.5 | 10.5 | 11.7 | 1.51 | **1.47** | **25.3** |
| Week 8 | 14 | 1.0% β-glucan | 21.0 | 11.0 | 12.2 | 1.58 |  |  |
| Week 8 | 14 | 1.0% β-glucan | 22.0 | 11.3 | 12.5 | 1.52 |  |  |
| Week 8 | 14 | 1.0% β-glucan | 34.0 | 13.4 | 14.3 | 1.41 |  |  |
| Week 8 | 14 | 1.0% β-glucan | 29.5 | 12.7 | 14.0 | 1.44 |  |  |
| Week 8 | 14 | 1.0% β-glucan | 25.5 | 11.8 | 12.8 | 1.55 |  |  |
| Week 8 | 14 | 1.0% β-glucan | 38.5 | 14.6 | 15.9 | 1.24 |  |  |
| Week 8 | 14 | 1.0% β-glucan | 26.0 | 12.2 | 13.3 | 1.43 |  |  |
| Week 8 | 14 | 1.0% β-glucan | 31.5 | 13.5 | 14.6 | 1.28 |  |  |
| Week 8 | 14 | 1.0% β-glucan | 17.5 | 10.5 | 11.7 | 1.51 |  |  |
| Week 8 | 14 | 1.0% β-glucan | 21.5 | 11.2 | 12.3 | 1.53 |  |  |
| Week 8 | 14 | 1.0% β-glucan | 18.0 | 10.8 | 12.0 | 1.43 |  |  |
| Week 8 | 14 | 1.0% β-glucan | 25.0 | 12.2 | 13.5 | 1.38 |  |  |
| Week 8 | 14 | 1.0% β-glucan | 31.5 | 12.7 | 13.7 | 1.54 |  |  |
| Week 8 | 14 | 1.0% β-glucan | 22.5 | 11.3 | 12.6 | 1.56 |  |  |
| Week 8 | 14 | 1.0% β-glucan | 36.0 | 13.6 | 15.0 | 1.43 |  |  |
| Week 8 | 14 | 1.0% β-glucan | 17.0 | 10.6 | 11.9 | 1.43 |  |  |
| Week 8 | 14 | 1.0% β-glucan | 20.0 | 11.2 | 12.4 | 1.42 |  |  |
| Week 8 | 14 | 1.0% β-glucan | 39.5 | 14.5 | 16.0 | 1.30 |  |  |
| Week 8 | 14 | 1.0% β-glucan | 29.5 | 12.9 | 14.1 | 1.37 |  |  |
| Week 8 | 14 | 1.0% β-glucan | 21.0 | 11.4 | 12.7 | 1.42 | **1.44** | **26.4** |
| Week 8 | 14 | 5.0% β-glucan | 24.0 | 11.9 | 13.1 | 1.42 |  |  |
| Week 8 | 14 | 5.0% β-glucan | 21.5 | 11.5 | 12.7 | 1.41 |  |  |
| Week 8 | 14 | 5.0% β-glucan | 27.5 | 12.3 | 13.8 | 1.48 |  |  |
| Week 8 | 14 | 5.0% β-glucan | 37.0 | 13.7 | 15.2 | 1.44 |  |  |
| Week 8 | 14 | 5.0% β-glucan | 18.5 | 10.9 | 11.9 | 1.43 |  |  |
| Week 8 | 14 | 5.0% β-glucan | 35.0 | 14.2 | 15.6 | 1.22 |  |  |
| Week 8 | 14 | 5.0% β-glucan | 39.5 | 14.0 | 15.4 | 1.44 |  |  |
| Week 8 | 14 | 5.0% β-glucan | 31.0 | 13.4 | 14.8 | 1.29 |  |  |
| Week 8 | 14 | 5.0% β-glucan | 24.0 | 11.8 | 13.1 | 1.46 |  |  |
| Week 8 | 14 | 5.0% β-glucan | 20.0 | 11.3 | 12.6 | 1.39 |  |  |
| Week 8 | 14 | 5.0% β-glucan | 24.5 | 12.2 | 13.5 | 1.35 |  |  |
| Week 8 | 14 | 5.0% β-glucan | 22.0 | 11.3 | 12.6 | 1.52 |  |  |
| Week 8 | 14 | 5.0% β-glucan | 27.0 | 12.3 | 13.5 | 1.45 |  |  |
| Week 8 | 14 | 5.0% β-glucan | 27.5 | 12.6 | 14.0 | 1.37 |  |  |
| Week 8 | 14 | 5.0% β-glucan | 17.0 | 10.5 | 11.6 | 1.47 |  |  |
| Week 8 | 14 | 5.0% β-glucan | 18.0 | 10.7 | 12.0 | 1.47 |  |  |
| Week 8 | 14 | 5.0% β-glucan | 24.5 | 12.0 | 13.5 | 1.42 |  |  |
| Week 8 | 14 | 5.0% β-glucan | 16.5 | 10.3 | 11.6 | 1.51 |  |  |
| Week 8 | 14 | 5.0% β-glucan | 18.0 | 10.7 | 11.9 | 1.47 |  |  |
| Week 8 | 14 | 5.0% β-glucan | 19.5 | 11.2 | 12.4 | 1.39 | **1.42** | **24.6** |
|  |  |  |  |  |  |  |  |  |
| Week 9 | 21 | 0% β-glucan (uninfected control) | 20.0 | 10.5 | 11.6 | 1.73 |  |  |
| Week 9 | 21 | 0% β-glucan (uninfected control) | 21.0 | 10.5 | 11.8 | 1.81 |  |  |
| Week 9 | 21 | 0% β-glucan (uninfected control) | 19.0 | 10.5 | 11.2 | 1.64 |  |  |
| Week 9 | 21 | 0% β-glucan (uninfected control) | 30.0 | 12.6 | 13.8 | 1.50 |  |  |
| Week 9 | 21 | 0% β-glucan (uninfected control) | 28.0 | 12.5 | 13.9 | 1.43 |  |  |
| Week 9 | 21 | 0% β-glucan (uninfected control) | 24.5 | 11.2 | 13.0 | 1.74 |  |  |
| Week 9 | 21 | 0% β-glucan (uninfected control) | 45.5 | 14.4 | 15.8 | 1.52 |  |  |
| Week 9 | 21 | 0% β-glucan (uninfected control) | 24.0 | 11.4 | 12.7 | 1.62 |  |  |
| Week 9 | 21 | 0% β-glucan (uninfected control) | 29.5 | 12.9 | 14.3 | 1.37 |  |  |
| Week 9 | 21 | 0% β-glucan (uninfected control) | 25.5 | 12.0 | 13.4 | 1.48 | **1.59** | **26.7** |
| Week 9 | 21 | 0% β-glucan | 21.5 | 11.0 | 12.2 | 1.62 |  |  |
| Week 9 | 21 | 0% β-glucan | 25.0 | 12.2 | 13.5 | 1.38 |  |  |
| Week 9 | 21 | 0% β-glucan | 26.0 | 12.7 | 13.8 | 1.27 |  |  |
| Week 9 | 21 | 0% β-glucan | 23.0 | 11.5 | 12.9 | 1.51 |  |  |
| Week 9 | 21 | 0% β-glucan | 25.5 | 12.0 | 13.2 | 1.48 |  |  |
| Week 9 | 21 | 0% β-glucan | 37.5 | 13.5 | 15.0 | 1.52 |  |  |
| Week 9 | 21 | 0% β-glucan | 30.0 | 13.1 | 14.0 | 1.33 |  |  |
| Week 9 | 21 | 0% β-glucan | 33.5 | 13.0 | 14.4 | 1.52 |  |  |
| Week 9 | 21 | 0% β-glucan | 28.5 | 12.6 | 13.5 | 1.42 |  |  |
| Week 9 | 21 | 0% β-glucan | 27.0 | 12.4 | 13.8 | 1.42 |  |  |
| Week 9 | 21 | 0% β-glucan | 29.0 | 12.7 | 14.0 | 1.42 |  |  |
| Week 9 | 21 | 0% β-glucan | 25.0 | 11.9 | 13.2 | 1.48 |  |  |
| Week 9 | 21 | 0% β-glucan | 35.5 | 13.3 | 14.6 | 1.51 |  |  |
| Week 9 | 21 | 0% β-glucan | 53.5 | 15.5 | 16.8 | 1.44 |  |  |
| Week 9 | 21 | 0% β-glucan | 25.0 | 11.7 | 12.9 | 1.56 |  |  |
| Week 9 | 21 | 0% β-glucan | 36.0 | 14.0 | 15.5 | 1.31 |  |  |
| Week 9 | 21 | 0% β-glucan | 32.0 | 13.2 | 14.5 | 1.39 |  |  |
| Week 9 | 21 | 0% β-glucan | 37.5 | 14.0 | 15.5 | 1.37 |  |  |
| Week 9 | 21 | 0% β-glucan | 24.0 | 11.0 | 12.6 | 1.80 |  |  |
| Week 9 | 21 | 0% β-glucan | 19.0 | 10.4 | 11.5 | 1.69 | **1.47** | **29.7** |
| Week 9 | 21 | 0.1% β-glucan | 28.5 | 12.3 | 13.6 | 1.53 |  |  |
| Week 9 | 21 | 0.1% β-glucan | 32.5 | 12.8 | 14.0 | 1.55 |  |  |
| Week 9 | 21 | 0.1% β-glucan | 19.0 | 10.6 | 12.1 | 1.60 |  |  |
| Week 9 | 21 | 0.1% β-glucan | 19.5 | 10.5 | 11.9 | 1.68 |  |  |
| Week 9 | 21 | 0.1% β-glucan | 31.0 | 12.5 | 13.6 | 1.59 |  |  |
| Week 9 | 21 | 0.1% β-glucan | 22.0 | 11.5 | 12.6 | 1.45 |  |  |
| Week 9 | 21 | 0.1% β-glucan | 39.5 | 13.5 | 15.0 | 1.61 |  |  |
| Week 9 | 21 | 0.1% β-glucan | 45.0 | 14.4 | 15.6 | 1.51 |  |  |
| Week 9 | 21 | 0.1% β-glucan | 25.5 | 11.7 | 13.0 | 1.59 |  |  |
| Week 9 | 21 | 0.1% β-glucan | 18.5 | 10.5 | 11.9 | 1.60 |  |  |
| Week 9 | 21 | 0.1% β-glucan | 25.0 | 11.4 | 12.5 | 1.69 |  |  |
| Week 9 | 21 | 0.1% β-glucan | 19.0 | 10.3 | 11.3 | 1.74 |  |  |
| Week 9 | 21 | 0.1% β-glucan | 21.5 | 11.2 | 12.4 | 1.53 |  |  |
| Week 9 | 21 | 0.1% β-glucan | 31.5 | 12.9 | 14.1 | 1.47 |  |  |
| Week 9 | 21 | 0.1% β-glucan | 28.0 | 12.1 | 13.4 | 1.58 |  |  |
| Week 9 | 21 | 0.1% β-glucan | 29.0 | 12.5 | 13.7 | 1.48 |  |  |
| Week 9 | 21 | 0.1% β-glucan | 27.5 | 12.0 | 13.3 | 1.59 |  |  |
| Week 9 | 21 | 0.1% β-glucan | 37.0 | 13.0 | 14.0 | 1.68 |  |  |
| Week 9 | 21 | 0.1% β-glucan | 27.5 | 11.7 | 13.2 | 1.72 |  |  |
| Week 9 | 21 | 0.1% β-glucan | 15.5 | 9.8 | 11.2 | 1.65 | **1.59** | **27.1** |
| Week 9 | 21 | 1.0% β-glucan | 22.5 | 11.2 | 12.5 | 1.60 |  |  |
| Week 9 | 21 | 1.0% β-glucan | 35.5 | 13.5 | 15.0 | 1.44 |  |  |
| Week 9 | 21 | 1.0% β-glucan | 25.0 | 11.8 | 13.0 | 1.52 |  |  |
| Week 9 | 21 | 1.0% β-glucan | 22.0 | 11.5 | 12.7 | 1.45 |  |  |
| Week 9 | 21 | 1.0% β-glucan | 24.0 | 11.6 | 12.8 | 1.54 |  |  |
| Week 9 | 21 | 1.0% β-glucan | 31.5 | 13.0 | 14.4 | 1.43 |  |  |
| Week 9 | 21 | 1.0% β-glucan | 31.0 | 12.7 | 14.0 | 1.51 |  |  |
| Week 9 | 21 | 1.0% β-glucan | 38.0 | 14.6 | 15.5 | 1.22 |  |  |
| Week 9 | 21 | 1.0% β-glucan | 18.5 | 10.4 | 11.5 | 1.64 |  |  |
| Week 9 | 21 | 1.0% β-glucan | 14.0 | 9.8 | 10.7 | 1.49 |  |  |
| Week 9 | 21 | 1.0% β-glucan | 19.5 | 10.8 | 12.0 | 1.55 |  |  |
| Week 9 | 21 | 1.0% β-glucan | 27.5 | 12.8 | 14.3 | 1.31 |  |  |
| Week 9 | 21 | 1.0% β-glucan | 17.5 | 10.7 | 11.7 | 1.43 |  |  |
| Week 9 | 21 | 1.0% β-glucan | 45.5 | 14.4 | 15.8 | 1.52 |  |  |
| Week 9 | 21 | 1.0% β-glucan | 27.0 | 12.1 | 13.3 | 1.52 |  |  |
| Week 9 | 21 | 1.0% β-glucan | 28.0 | 12.5 | 13.6 | 1.43 |  |  |
| Week 9 | 21 | 1.0% β-glucan | 29.5 | 12.7 | 14.1 | 1.44 |  |  |
| Week 9 | 21 | 1.0% β-glucan | 19.5 | 10.8 | 12.0 | 1.55 |  |  |
| Week 9 | 21 | 1.0% β-glucan | 28.0 | 12.4 | 13.5 | 1.47 |  |  |
| Week 9 | 21 | 1.0% β-glucan | 23.5 | 11.7 | 13.0 | 1.47 | **1.48** | **26.4** |
| Week 9 | 21 | 5.0% β-glucan | 19.0 | 10.8 | 12.1 | 1.51 |  |  |
| Week 9 | 21 | 5.0% β-glucan | 32.5 | 13.0 | 14.5 | 1.48 |  |  |
| Week 9 | 21 | 5.0% β-glucan | 30.5 | 12.8 | 14.0 | 1.45 |  |  |
| Week 9 | 21 | 5.0% β-glucan | 18.0 | 10.5 | 11.8 | 1.55 |  |  |
| Week 9 | 21 | 5.0% β-glucan | 21.0 | 11.2 | 12.4 | 1.49 |  |  |
| Week 9 | 21 | 5.0% β-glucan | 31.5 | 13.0 | 14.2 | 1.43 |  |  |
| Week 9 | 21 | 5.0% β-glucan | 30.5 | 12.8 | 14.0 | 1.45 |  |  |
| Week 9 | 21 | 5.0% β-glucan | 32.0 | 13.0 | 14.5 | 1.46 |  |  |
| Week 9 | 21 | 5.0% β-glucan | 20.0 | 11.2 | 12.5 | 1.42 |  |  |
| Week 9 | 21 | 5.0% β-glucan | 26.5 | 12.3 | 14.0 | 1.42 |  |  |
| Week 9 | 21 | 5.0% β-glucan | 27.0 | 12.2 | 13.5 | 1.49 |  |  |
| Week 9 | 21 | 5.0% β-glucan | 34.5 | 13.0 | 14.5 | 1.57 |  |  |
| Week 9 | 21 | 5.0% β-glucan | 34.5 | 13.4 | 14.8 | 1.43 |  |  |
| Week 9 | 21 | 5.0% β-glucan | 18.5 | 10.9 | 12.2 | 1.43 |  |  |
| Week 9 | 21 | 5.0% β-glucan | 33.5 | 13.5 | 14.7 | 1.36 |  |  |
| Week 9 | 21 | 5.0% β-glucan | 31.5 | 13.0 | 14.4 | 1.43 |  |  |
| Week 9 | 21 | 5.0% β-glucan | 44.5 | 14.8 | 16.0 | 1.37 |  |  |
| Week 9 | 21 | 5.0% β-glucan | 25.5 | 12.4 | 13.6 | 1.34 |  |  |
| Week 9 | 21 | 5.0% β-glucan | 22.5 | 11.4 | 12.5 | 1.52 |  |  |
| Week 9 | 21 | 5.0% β-glucan | 15.5 | 10.0 | 10.9 | 1.55 | **1.46** | **27.5** |
|  |  |  |  |  |  |  |  |  |
| Week 10 | 28 | 0% β-glucan (uninfected control) | 30.0 | 12.3 | 13.8 | 1.61 |  |  |
| Week 10 | 28 | 0% β-glucan (uninfected control) | 26.5 | 12.0 | 13.3 | 1.53 |  |  |
| Week 10 | 28 | 0% β-glucan (uninfected control) | 19.5 | 10.5 | 11.6 | 1.68 |  |  |
| Week 10 | 28 | 0% β-glucan (uninfected control) | 41.5 | 14.2 | 15.4 | 1.45 |  |  |
| Week 10 | 28 | 0% β-glucan (uninfected control) | 25.0 | 11.8 | 13.1 | 1.52 |  |  |
| Week 10 | 28 | 0% β-glucan (uninfected control) | 24.5 | 11.3 | 12.6 | 1.70 |  |  |
| Week 10 | 28 | 0% β-glucan (uninfected control) | 25.0 | 11.7 | 13.0 | 1.56 |  |  |
| Week 10 | 28 | 0% β-glucan (uninfected control) | 23.5 | 11.5 | 12.8 | 1.55 |  |  |
| Week 10 | 28 | 0% β-glucan (uninfected control) | 26.0 | 11.6 | 13.0 | 1.67 |  |  |
| Week 10 | 28 | 0% β-glucan (uninfected control) | 32.5 | 12.6 | 14.1 | 1.62 | **1.59** | **27.4** |
| Week 10 | 28 | 0% β-glucan | 28.0 | 12.1 | 13.4 | 1.58 |  |  |
| Week 10 | 28 | 0% β-glucan | 29.5 | 12.3 | 13.7 | 1.59 |  |  |
| Week 10 | 28 | 0% β-glucan | 47.5 | 14.3 | 16.0 | 1.62 |  |  |
| Week 10 | 28 | 0% β-glucan | 23.5 | 11.6 | 12.8 | 1.51 |  |  |
| Week 10 | 28 | 0% β-glucan | 27.5 | 12.3 | 13.6 | 1.48 |  |  |
| Week 10 | 28 | 0% β-glucan | 26.5 | 12.4 | 13.3 | 1.39 |  |  |
| Week 10 | 28 | 0% β-glucan | 36.0 | 13.5 | 14.7 | 1.46 |  |  |
| Week 10 | 28 | 0% β-glucan | 29.0 | 12.3 | 13.5 | 1.56 |  |  |
| Week 10 | 28 | 0% β-glucan | 31.0 | 12.7 | 13.9 | 1.51 |  |  |
| Week 10 | 28 | 0% β-glucan | 29.5 | 12.7 | 14.0 | 1.44 |  |  |
| Week 10 | 28 | 0% β-glucan | 25.0 | 11.9 | 13.0 | 1.48 |  |  |
| Week 10 | 28 | 0% β-glucan | 27.5 | 12.6 | 13.7 | 1.37 |  |  |
| Week 10 | 28 | 0% β-glucan | 32.5 | 13.2 | 14.6 | 1.41 |  |  |
| Week 10 | 28 | 0% β-glucan | 24.0 | 11.5 | 12.7 | 1.58 |  |  |
| Week 10 | 28 | 0% β-glucan | 50.0 | 15.4 | 16.2 | 1.37 |  |  |
| Week 10 | 28 | 0% β-glucan | 20.0 | 10.8 | 12.0 | 1.59 |  |  |
| Week 10 | 28 | 0% β-glucan | 23.0 | 11.3 | 12.7 | 1.59 |  |  |
| Week 10 | 28 | 0% β-glucan | 21.5 | 11.2 | 12.3 | 1.53 |  |  |
| Week 10 | 28 | 0% β-glucan | 23.0 | 11.4 | 12.8 | 1.55 |  |  |
| Week 10 | 28 | 0% β-glucan | 17.5 | 9.8 | 10.8 | 1.86 |  |  |
| Week 10 | 28 | 0% β-glucan | 33.5 | 13.2 | 14.7 | 1.46 |  |  |
| Week 10 | 28 | 0% β-glucan | 22.5 | 11.2 | 12.1 | 1.60 |  |  |
| Week 10 | 28 | 0% β-glucan | 23.5 | 11.4 | 12.7 | 1.59 |  |  |
| Week 10 | 28 | 0% β-glucan | 27.0 | 11.8 | 13.0 | 1.64 |  |  |
| Week 10 | 28 | 0% β-glucan | 20.0 | 11.0 | 11.9 | 1.50 |  |  |
| Week 10 | 28 | 0% β-glucan | 21.5 | 10.9 | 12.1 | 1.66 |  |  |
| Week 10 | 28 | 0% β-glucan | 42.0 | 14.0 | 15.0 | 1.53 |  |  |
| Week 10 | 28 | 0% β-glucan | 21.5 | 11.2 | 12.4 | 1.53 |  |  |
| Week 10 | 28 | 0% β-glucan | 54.0 | 15.7 | 16.9 | 1.40 |  |  |
| Week 10 | 28 | 0% β-glucan | 20.5 | 10.4 | 11.6 | 1.82 | **1.54** | **28.6** |
| Week 10 | 28 | 0.1% β-glucan | 38.0 | 13.7 | 15.1 | 1.48 |  |  |
| Week 10 | 28 | 0.1% β-glucan | 25.5 | 11.9 | 13.2 | 1.51 |  |  |
| Week 10 | 28 | 0.1% β-glucan | 37.0 | 13.4 | 14.9 | 1.54 |  |  |
| Week 10 | 28 | 0.1% β-glucan | 21.5 | 11.1 | 12.2 | 1.57 |  |  |
| Week 10 | 28 | 0.1% β-glucan | 21.5 | 11.0 | 12.2 | 1.62 |  |  |
| Week 10 | 28 | 0.1% β-glucan | 29.0 | 12.0 | 13.2 | 1.68 |  |  |
| Week 10 | 28 | 0.1% β-glucan | 17.0 | 10.0 | 11.1 | 1.70 |  |  |
| Week 10 | 28 | 0.1% β-glucan | 23.0 | 11.4 | 12.5 | 1.55 |  |  |
| Week 10 | 28 | 0.1% β-glucan | 18.0 | 10.3 | 11.2 | 1.65 |  |  |
| Week 10 | 28 | 0.1% β-glucan | 24.0 | 11.2 | 12.4 | 1.71 |  |  |
| Week 10 | 28 | 0.1% β-glucan | 27.5 | 12.5 | 13.7 | 1.41 |  |  |
| Week 10 | 28 | 0.1% β-glucan | 29.0 | 12.4 | 13.7 | 1.52 |  |  |
| Week 10 | 28 | 0.1% β-glucan | 39.5 | 14.2 | 15.4 | 1.38 |  |  |
| Week 10 | 28 | 0.1% β-glucan | 42.5 | 14.0 | 15.2 | 1.55 |  |  |
| Week 10 | 28 | 0.1% β-glucan | 26.0 | 12.0 | 12.9 | 1.50 |  |  |
| Week 10 | 28 | 0.1% β-glucan | 32.0 | 12.8 | 14.0 | 1.53 |  |  |
| Week 10 | 28 | 0.1% β-glucan | 27.5 | 12.3 | 13.7 | 1.48 |  |  |
| Week 10 | 28 | 0.1% β-glucan | 17.5 | 10.3 | 11.2 | 1.60 |  |  |
| Week 10 | 28 | 0.1% β-glucan | 32.5 | 12.4 | 13.8 | 1.70 |  |  |
| Week 10 | 28 | 0.1% β-glucan | 23.0 | 11.1 | 12.4 | 1.68 |  |  |
| Week 10 | 28 | 0.1% β-glucan | 41.0 | 14.0 | 14.9 | 1.49 |  |  |
| Week 10 | 28 | 0.1% β-glucan | 41.0 | 13.7 | 15.0 | 1.59 |  |  |
| Week 10 | 28 | 0.1% β-glucan | 40.5 | 14.1 | 15.2 | 1.44 |  |  |
| Week 10 | 28 | 0.1% β-glucan | 27.0 | 12.4 | 13.5 | 1.42 |  |  |
| Week 10 | 28 | 0.1% β-glucan | 24.5 | 11.2 | 12.7 | 1.74 |  |  |
| Week 10 | 28 | 0.1% β-glucan | 32.0 | 12.2 | 13.4 | 1.76 |  |  |
| Week 10 | 28 | 0.1% β-glucan | 31.0 | 12.5 | 13.7 | 1.59 |  |  |
| Week 10 | 28 | 0.1% β-glucan | 28.5 | 12.5 | 13.8 | 1.46 |  |  |
| Week 10 | 28 | 0.1% β-glucan | 47.0 | 14.6 | 16.0 | 1.51 |  |  |
| Week 10 | 28 | 0.1% β-glucan | 19.5 | 10.8 | 12.0 | 1.55 | **1.56** | **29.5** |
| Week 10 | 28 | 1.0% β-glucan | 18.0 | 10.4 | 11.7 | 1.60 |  |  |
| Week 10 | 28 | 1.0% β-glucan | 24.0 | 11.5 | 12.7 | 1.58 |  |  |
| Week 10 | 28 | 1.0% β-glucan | 17.0 | 10.4 | 11.4 | 1.51 |  |  |
| Week 10 | 28 | 1.0% β-glucan | 16.0 | 9.9 | 11.0 | 1.65 |  |  |
| Week 10 | 28 | 1.0% β-glucan | 39.0 | 13.8 | 15.0 | 1.48 |  |  |
| Week 10 | 28 | 1.0% β-glucan | 20.0 | 10.7 | 11.9 | 1.63 |  |  |
| Week 10 | 28 | 1.0% β-glucan | 37.0 | 13.4 | 14.5 | 1.54 |  |  |
| Week 10 | 28 | 1.0% β-glucan | 33.5 | 13.2 | 14.3 | 1.46 |  |  |
| Week 10 | 28 | 1.0% β-glucan | 17.5 | 10.5 | 11.7 | 1.51 |  |  |
| Week 10 | 28 | 1.0% β-glucan | 43.5 | 14.0 | 15.4 | 1.59 |  |  |
| Week 10 | 28 | 1.0% β-glucan | 26.0 | 12.4 | 13.2 | 1.36 |  |  |
| Week 10 | 28 | 1.0% β-glucan | 34.5 | 13.4 | 14.3 | 1.43 |  |  |
| Week 10 | 28 | 1.0% β-glucan | 31.0 | 13.3 | 14.2 | 1.32 |  |  |
| Week 10 | 28 | 1.0% β-glucan | 27.5 | 12.5 | 13.6 | 1.41 |  |  |
| Week 10 | 28 | 1.0% β-glucan | 18.5 | 10.5 | 11.6 | 1.60 |  |  |
| Week 10 | 28 | 1.0% β-glucan | 31.0 | 12.9 | 14.3 | 1.44 |  |  |
| Week 10 | 28 | 1.0% β-glucan | 21.0 | 11.0 | 12.0 | 1.58 |  |  |
| Week 10 | 28 | 1.0% β-glucan | 29.5 | 12.7 | 14.1 | 1.44 |  |  |
| Week 10 | 28 | 1.0% β-glucan | 26.0 | 12.1 | 13.5 | 1.47 |  |  |
| Week 10 | 28 | 1.0% β-glucan | 35.0 | 13.6 | 14.8 | 1.39 |  |  |
| Week 10 | 28 | 1.0% β-glucan | 32.0 | 12.5 | 13.7 | 1.64 |  |  |
| Week 10 | 28 | 1.0% β-glucan | 24.0 | 11.4 | 12.6 | 1.62 |  |  |
| Week 10 | 28 | 1.0% β-glucan | 50.0 | 15.0 | 16.1 | 1.48 |  |  |
| Week 10 | 28 | 1.0% β-glucan | 30.5 | 12.7 | 13.9 | 1.49 |  |  |
| Week 10 | 28 | 1.0% β-glucan | 32.5 | 12.9 | 14.9 | 1.51 |  |  |
| Week 10 | 28 | 1.0% β-glucan | 17.5 | 10.2 | 11.4 | 1.65 |  |  |
| Week 10 | 28 | 1.0% β-glucan | 28.5 | 12.3 | 13.6 | 1.53 |  |  |
| Week 10 | 28 | 1.0% β-glucan | 26.0 | 12.3 | 13.5 | 1.40 |  |  |
| Week 10 | 28 | 1.0% β-glucan | 25.0 | 11.9 | 13.2 | 1.48 |  |  |
| Week 10 | 28 | 1.0% β-glucan | 16.0 | 10.0 | 11.1 | 1.60 | **1.51** | **27.6** |
| Week 10 | 28 | 5.0% β-glucan | 50.5 | 14.9 | 16.9 | 1.53 |  |  |
| Week 10 | 28 | 5.0% β-glucan | 25.0 | 11.7 | 12.8 | 1.56 |  |  |
| Week 10 | 28 | 5.0% β-glucan | 21.5 | 11.2 | 12.5 | 1.53 |  |  |
| Week 10 | 28 | 5.0% β-glucan | 27.0 | 12.2 | 13.4 | 1.49 |  |  |
| Week 10 | 28 | 5.0% β-glucan | 18.5 | 10.5 | 11.7 | 1.60 |  |  |
| Week 10 | 28 | 5.0% β-glucan | 51.0 | 15.7 | 16.5 | 1.32 |  |  |
| Week 10 | 28 | 5.0% β-glucan | 46.5 | 14.7 | 16.2 | 1.46 |  |  |
| Week 10 | 28 | 5.0% β-glucan | 23.5 | 11.5 | 12.7 | 1.55 |  |  |
| Week 10 | 28 | 5.0% β-glucan | 34.5 | 13.4 | 14.7 | 1.43 |  |  |
| Week 10 | 28 | 5.0% β-glucan | 16.0 | 10.0 | 11.1 | 1.60 |  |  |
| Week 10 | 28 | 5.0% β-glucan | 26.5 | 12.2 | 13.5 | 1.46 |  |  |
| Week 10 | 28 | 5.0% β-glucan | 30.5 | 12.4 | 13.7 | 1.60 |  |  |
| Week 10 | 28 | 5.0% β-glucan | 33.5 | 13.3 | 14.5 | 1.42 |  |  |
| Week 10 | 28 | 5.0% β-glucan | 23.5 | 11.4 | 12.8 | 1.59 |  |  |
| Week 10 | 28 | 5.0% β-glucan | 33.0 | 13.7 | 15.1 | 1.28 |  |  |
| Week 10 | 28 | 5.0% β-glucan | 18.0 | 10.2 | 11.2 | 1.70 |  |  |
| Week 10 | 28 | 5.0% β-glucan | 20.5 | 11.3 | 12.4 | 1.42 |  |  |
| Week 10 | 28 | 5.0% β-glucan | 28.5 | 12.5 | 13.7 | 1.46 |  |  |
| Week 10 | 28 | 5.0% β-glucan | 39.5 | 13.9 | 15.0 | 1.47 |  |  |
| Week 10 | 28 | 5.0% β-glucan | 34.0 | 13.6 | 14.7 | 1.35 |  |  |
| Week 10 | 28 | 5.0% β-glucan | 31.0 | 12.7 | 14.0 | 1.51 |  |  |
| Week 10 | 28 | 5.0% β-glucan | 39.0 | 13.6 | 14.9 | 1.55 |  |  |
| Week 10 | 28 | 5.0% β-glucan | 31.0 | 12.9 | 14.2 | 1.44 |  |  |
| Week 10 | 28 | 5.0% β-glucan | 28.0 | 12.5 | 13.6 | 1.43 |  |  |
| Week 10 | 28 | 5.0% β-glucan | 18.0 | 10.7 | 12.0 | 1.47 |  |  |
| Week 10 | 28 | 5.0% β-glucan | 28.5 | 12.4 | 13.6 | 1.49 |  |  |
| Week 10 | 28 | 5.0% β-glucan | 24.5 | 11.5 | 12.8 | 1.61 |  |  |
| Week 10 | 28 | 5.0% β-glucan | 21.0 | 11.4 | 12.5 | 1.42 |  |  |
| Week 10 | 28 | 5.0% β-glucan | 28.5 | 12.5 | 13.7 | 1.46 |  |  |
| Week 10 | 28 | 5.0% β-glucan | - | - | - | - | **1.49** | **29.3** |

Additional file 1: Figure S6. Average weight of the fish per treatment over the course of the experiment.


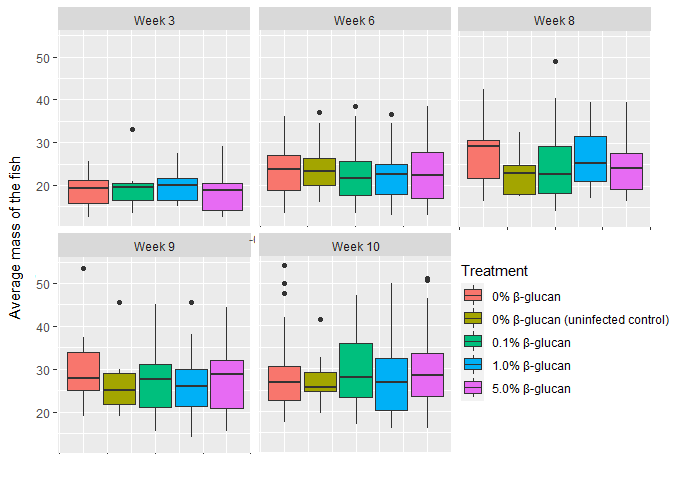


Additional file 1: Figure S7. Average Fulton's condition factor (K) of the fish per treatment over the course of the experiment.


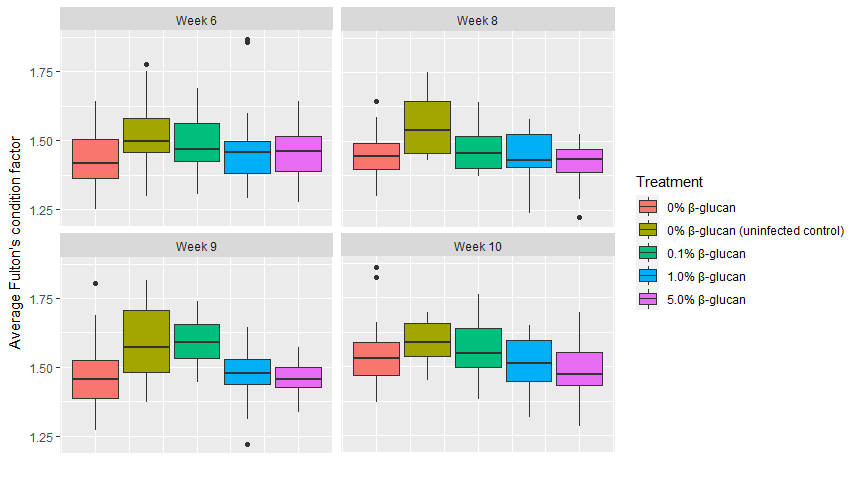


Additional file 1: Table S8. All metabolites measured in the serum of the fish for all investigated groups.

| **Metabolites** |  |  |  |  |
| --- | --- | --- | --- | --- |
| **FIA compounds** |  |  |  |  |
| Carnitine (free) |  |  |  |  |
| Acetylcarnitine |  |  |  |  |
| Propionylcarnitine |  |  |  |  |
| Propenoylcarnitine |  |  |  |  |
| Hydroxypropionylcarnitine |  |  |  |  |
| Butyrylcarnitine / Isobutyrylcarnitine |  |  |  |  |
| Butenoylcarnitine |  |  |  |  |
| Hydroxybutyrylcarnitine (Malonylcarnitine) |  |  |  |  |
| Isovalerylcarnitine / 2-Methylbutyrylcarnitine / Valerylcarnitine |  |  |  |  |
| Tiglylcarnitine / 3-Methyl-crotonylcarnitine |  |  |  |  |
| Glutaconylcarnitine / Mesaconylcarnitine |  |  |  |  |
| Glutarylcarnitine (Hydroxyhexanoylcarnitine [= Hydroxycaproylcarnitine]) |  |  |  |  |
| Methylglutarylcarnitine |  |  |  |  |
| Hydroxyisovalerylcarnitine / Hydroxy-2-methylbutyryl / Hydroxyvalerylcarnitine (Methylmalonylcarnitine) |  |  |  |  |
| Hexanoylcarnitine [= Caproylcarnitine] (Fumarylcarnitine) |  |  |  |  |
| Hexenoylcarnitine |  |  |  |  |
| Pimelylcarnitine |  |  |  |  |
| Octanoylcarnitine [= Caprylylcarnitine] |  |  |  |  |
| Nonanoylcarnitine [= Pelargonylcarnitine] |  |  |  |  |
| Decanoylcarnitine [= Caprylcarnitine] |  |  |  |  |
| Decenoylcarnitine |  |  |  |  |
| Decadienoylcarnitine |  |  |  |  |
| Dodecanoylcarnitine [= Laurylcarnitine] |  |  |  |  |
| Dodecenoylcarnitine |  |  |  |  |
| Dodecanedioylcarnitine |  |  |  |  |
| Tetradecanoylcarnitine [= Myristylcarnitine] |  |  |  |  |
| Tetradecenoylcarnitine [= Myristoleylcarnitine] |  |  |  |  |
| Hydroxytetradecenoylcarnitine [= Hydroxymyristoleylcarnitine] |  |  |  |  |
| Tetradecadienoylcarnitine |  |  |  |  |
| Hydroxytetradecadienoylcarnitine |  |  |  |  |
| Hexadecanoylcarnitine [= Palmitoylcarnitine] |  |  |  |  |
| Hexadecenoylcarnitine [= Palmitoleylcarnitine] |  |  |  |  |
| Hydroxyhexadecenoylcarnitine [= Hydroxypalmitoleylcarnitine] |  |  |  |  |
| Hexadecadienoylcarnitine |  |  |  |  |
| Hydroxyhexadecadienoylcarnitine |  |  |  |  |
| Hydroxyhexadecanolycarnitine [= Hydroxypalmitoylcarnitine] |  |  |  |  |
| Octadecanoylcarnitine [= Stearylcarnitine] |  |  |  |  |
| Octadecenoylcarnitine [= Oleylcarnitine] |  |  |  |  |
| Hydroxyoctadecenoylcarnitine [= Hydroxyoleylcarnitine] |  |  |  |  |
| Octadecadienoylcarnitine [= Linoleylcarnitine] |  |  |  |  |
| Lysophosphatidylcholine with acyl residue C16:1 |  |  |  |  |
| Lysophosphatidylcholine with acyl residue C17:0 |  |  |  |  |
| Lysophosphatidylcholine with acyl residue C18:0 |  |  |  |  |
| Lysophosphatidylcholine with acyl residue C18:1 |  |  |  |  |
| Lysophosphatidylcholine with acyl residue C18:2 |  |  |  |  |
| Lysophosphatidylcholine with acyl residue C20:3 |  |  |  |  |
| Lysophosphatidylcholine with acyl residue C20:4 |  |  |  |  |
| Lysophosphatidylcholine with acyl residue C24:0 |  |  |  |  |
| Lysophosphatidylcholine with acyl residue C26:0 |  |  |  |  |
| Lysophosphatidylcholine with acyl residue C26:1 |  |  |  |  |
| Lysophosphatidylcholine with acyl residue C28:0 |  |  |  |  |
| Lysophosphatidylcholine with acyl residue C28:1 |  |  |  |  |
| Lysophosphatidylcholine with acyl residue C30:0 |  |  |  |  |
| Phosphatidylcholine with diacyl residue sum C30:2 |  |  |  |  |
| Phosphatidylcholine with diacyl residue sum C32:0 |  |  |  |  |
| Phosphatidylcholine with diacyl residue sum C32:1 |  |  |  |  |
| Phosphatidylcholine with diacyl residue sum C32:2 |  |  |  |  |
| Phosphatidylcholine with diacyl residue sum C32:3 |  |  |  |  |
| Phosphatidylcholine with diacyl residue sum C34:1 |  |  |  |  |
| Phosphatidylcholine with diacyl residue sum C34:2 |  |  |  |  |
| Phosphatidylcholine with diacyl residue sum C34:3 |  |  |  |  |
| Phosphatidylcholine with diacyl residue sum C34:4 |  |  |  |  |
| Phosphatidylcholine with diacyl residue sum C36:0 |  |  |  |  |
| Phosphatidylcholine with diacyl residue sum C36:1 |  |  |  |  |
| Phosphatidylcholine with diacyl residue sum C36:2 |  |  |  |  |
| Phosphatidylcholine with diacyl residue sum C36:3 |  |  |  |  |
| Phosphatidylcholine with diacyl residue sum C36:4 |  |  |  |  |
| Phosphatidylcholine with diacyl residue sum C36:5 |  |  |  |  |
| Phosphatidylcholine with diacyl residue sum C36:6 |  |  |  |  |
| Phosphatidylcholine with diacyl residue sum C38:0 |  |  |  |  |
| Phosphatidylcholine with diacyl residue sum C38:1 |  |  |  |  |
| Phosphatidylcholine with diacyl residue sum C38:3 |  |  |  |  |
| Phosphatidylcholine with diacyl residue sum C38:4 |  |  |  |  |
| Phosphatidylcholine with diacyl residue sum C38:5 |  |  |  |  |
| Phosphatidylcholine with diacyl residue sum C38:6 |  |  |  |  |
| Phosphatidylcholine with diacyl residue sum C40:1 |  |  |  |  |
| Phosphatidylcholine with diacyl residue sum C40:2 |  |  |  |  |
| Phosphatidylcholine with diacyl residue sum C40:3 |  |  |  |  |
| Phosphatidylcholine with diacyl residue sum C40:4 |  |  |  |  |
| Phosphatidylcholine with diacyl residue sum C40:5 |  |  |  |  |
| Phosphatidylcholine with diacyl residue sum C40:6 |  |  |  |  |
| Phosphatidylcholine with diacyl residue sum C42:0 |  |  |  |  |
| Phosphatidylcholine with diacyl residue sum C42:1 |  |  |  |  |
| Phosphatidylcholine with diacyl residue sum C42:2 |  |  |  |  |
| Phosphatidylcholine with diacyl residue sum C42:4 |  |  |  |  |
| Phosphatidylcholine with diacyl residue sum C42:5 |  |  |  |  |
| Phosphatidylcholine with diacyl residue sum C42:6 |  |  |  |  |
| Phosphatidylcholine with acyl-alkyl residue sum C30:0 |  |  |  |  |
| Phosphatidylcholine with acyl-alkyl residue sum C30:1 |  |  |  |  |
| Phosphatidylcholine with acyl-alkyl residue sum C30:2 |  |  |  |  |
| Phosphatidylcholine with acyl-alkyl residue sum C32:1 |  |  |  |  |
| Phosphatidylcholine with acyl-alkyl residue sum C32:2 |  |  |  |  |
| Phosphatidylcholine with acyl-alkyl residue sum C34:0 |  |  |  |  |
| Phosphatidylcholine with acyl-alkyl residue sum C34:1 |  |  |  |  |
| Phosphatidylcholine with acyl-alkyl residue sum C34:2 |  |  |  |  |
| Phosphatidylcholine with acyl-alkyl residue sum C34:3 |  |  |  |  |
| Phosphatidylcholine with acyl-alkyl residue sum C36:0 |  |  |  |  |
| Phosphatidylcholine with acyl-alkyl residue sum C36:1 |  |  |  |  |
| Phosphatidylcholine with acyl-alkyl residue sum C36:2 |  |  |  |  |
| Phosphatidylcholine with acyl-alkyl residue sum C36:3 |  |  |  |  |
| Phosphatidylcholine with acyl-alkyl residue sum C36:4 |  |  |  |  |
| Phosphatidylcholine with acyl-alkyl residue sum C36:5 |  |  |  |  |
| Phosphatidylcholine with acyl-alkyl residue sum C38:0 |  |  |  |  |
| Phosphatidylcholine with acyl-alkyl residue sum C38:1 |  |  |  |  |
| Phosphatidylcholine with acyl-alkyl residue sum C38:2 |  |  |  |  |
| Phosphatidylcholine with acyl-alkyl residue sum C38:3 |  |  |  |  |
| Phosphatidylcholine with acyl-alkyl residue sum C38:4 |  |  |  |  |
| Phosphatidylcholine with acyl-alkyl residue sum C38:5 |  |  |  |  |
| Phosphatidylcholine with acyl-alkyl residue sum C38:6 |  |  |  |  |
| Phosphatidylcholine with acyl-alkyl residue sum C40:0 |  |  |  |  |
| Phosphatidylcholine with acyl-alkyl residue sum C40:1 |  |  |  |  |
| Phosphatidylcholine with acyl-alkyl residue sum C40:2 |  |  |  |  |
| Phosphatidylcholine with acyl-alkyl residue sum C40:3 |  |  |  |  |
| Phosphatidylcholine with acyl-alkyl residue sum C40:4 |  |  |  |  |
| Phosphatidylcholine with acyl-alkyl residue sum C40:5 |  |  |  |  |
| Phosphatidylcholine with acyl-alkyl residue sum C40:6 |  |  |  |  |
| Phosphatidylcholine with acyl-alkyl residue sum C42:0 |  |  |  |  |
| Phosphatidylcholine with acyl-alkyl residue sum C42:1 |  |  |  |  |
| Phosphatidylcholine with acyl-alkyl residue sum C42:2 |  |  |  |  |
| Phosphatidylcholine with acyl-alkyl residue sum C42:3 |  |  |  |  |
| Phosphatidylcholine with acyl-alkyl residue sum C42:4 |  |  |  |  |
| Phosphatidylcholine with acyl-alkyl residue sum C42:5 |  |  |  |  |
| Phosphatidylcholine with acyl-alkyl residue sum C44:3 |  |  |  |  |
| Phosphatidylcholine with acyl-alkyl residue sum C44:4 |  |  |  |  |
| Phosphatidylcholine with acyl-alkyl residue sum C44:5 |  |  |  |  |
| Phosphatidylcholine with acyl-alkyl residue sum C44:6 |  |  |  |  |
| Hydroxysphingomyelin with acyl residue sum C14:1 |  |  |  |  |
| Hydroxysphingomyelin with acyl residue sum C16:1 |  |  |  |  |
| Hydroxysphingomyelin with acyl residue sum C22:1 |  |  |  |  |
| Hydroxysphingomyelin with acyl residue sum C22:2 |  |  |  |  |
| Hydroxysphingomyelin with acyl residue sum C24:1 |  |  |  |  |
| Sphingomyelin with acyl residue sum C16:0 |  |  |  |  |
| Sphingomyelin with acyl residue sum C16:1 |  |  |  |  |
| Sphingomyelin with acyl residue sum C18:0 |  |  |  |  |
| Sphingomyelin with acyl residue sum C18:1 |  |  |  |  |
| Sphingomyelin with acyl residue sum C20:2 |  |  |  |  |
| Sphingomyelin with acyl residue sum C22:3 |  |  |  |  |
| Sphingomyelin with acyl residue sum C24:0 |  |  |  |  |
| Sphingomyelin with acyl residue sum C24:1 |  |  |  |  |
| Sphingomyelin with acyl residue sum C26:0 |  |  |  |  |
| Sphingomyelin with acyl residue sum C26:1 |  |  |  |  |
| Sugars |  |  |  |  |
| (C2+C3) / C0 |  |  |  |  |
| C2 / C0 |  |  |  |  |
| CPT-I ratio |  |  |  |  |
| MUFA (PC) |  |  |  |  |
| MUFA (PC) / SFA (PC) |  |  |  |  |
| PUFA (PC) |  |  |  |  |
| PUFA (PC) / MUFA (PC) |  |  |  |  |
| PUFA (PC) / SFA (PC) |  |  |  |  |
| SFA (PC) |  |  |  |  |
| Total (PC+SM) |  |  |  |  |
| Total AC / C0 |  |  |  |  |
| Total AC-DC / Total AC |  |  |  |  |
| Total AC-OH / Total AC |  |  |  |  |
| Total lysoPC |  |  |  |  |
| Total lysoPC / Total PC |  |  |  |  |
| Total PC |  |  |  |  |
| Total PC aa |  |  |  |  |
| Total PC ae |  |  |  |  |
| Total SM |  |  |  |  |
| Total SM / Total (SM+PC) |  |  |  |  |
| Total SM / Total PC |  |  |  |  |
| Total SM-non OH |  |  |  |  |
| Total SM-OH |  |  |  |  |
| Total SM-OH / Total SM-non OH |  |  |  |  |
| **LC compounds** |  |  |  |  |
| Alanine |  |  |  |  |
| Arginine |  |  |  |  |
| Asparagine |  |  |  |  |
| Aspartic acid |  |  |  |  |
| Cysteine |  |  |  |  |
| Glutamine |  |  |  |  |
| Glutamic acid |  |  |  |  |
| Glycine |  |  |  |  |
| Histidine |  |  |  |  |
| Isoleucine |  |  |  |  |
| Leucine |  |  |  |  |
| Lysine |  |  |  |  |
| Methionine |  |  |  |  |
| Ornithine |  |  |  |  |
| Phenylalanine |  |  |  |  |
| Proline |  |  |  |  |
| Serine |  |  |  |  |
| Threonine |  |  |  |  |
| Tryptophan |  |  |  |  |
| Tyrosine |  |  |  |  |
| Valine |  |  |  |  |
| Ac-Orn |  |  |  |  |
| ADMA |  |  |  |  |
| Alpha-AAA |  |  |  |  |
| c4-OH-Pro |  |  |  |  |
| Creatinine |  |  |  |  |
| DOPA |  |  |  |  |
| Histamine |  |  |  |  |
| Met-SO |  |  |  |  |
| Nitrotyrosine |  |  |  |  |
| Putrescine |  |  |  |  |
| SDMA |  |  |  |  |
| Serotonin |  |  |  |  |
| Spermidine |  |  |  |  |
| t4-OH-Pro |  |  |  |  |
| Taurine |  |  |  |  |
| Total DMA |  |  |  |  |
| AAA |  |  |  |  |
| ADMA-Arginine |  |  |  |  |
| BCAA |  |  |  |  |
| Cit-Arg |  |  |  |  |
| Cit-Orn |  |  |  |  |
| Essential Amino acids |  |  |  |  |
| Fisher ratio |  |  |  |  |
| Glucogenic AA |  |  |  |  |
| Met-SO-Met |  |  |  |  |
| Non-essential Amino acids |  |  |  |  |
| Orn-Arg |  |  |  |  |
| Putrescine-Orn |  |  |  |  |
| SDMA-rp |  |  |  |  |
| Serotonin-rp |  |  |  |  |
| Spermidine-Putrescine |  |  |  |  |
| Spermine-Spermidine |  |  |  |  |
| Total Amino acids |  |  |  |  |
| Total DMA-Arg |  |  |  |  |
| Tyr-Phe |  |  |  |  |

Additional file 1: Table S9. Metabolites measured at significantly levels between treated fish and the control. Arrows indicate if the metabolite were upregulated (↑) or downregulated (↓) relative to the control without β-glucan treatment. Lack of significant change is indicated by “No”.

| Metabolite | 0.1% | 1.0% | 5.0% | p-value |
| --- | --- | --- | --- | --- |
| **FIA compounds** |  |  |  |  |
| Lysophosphatidylcholine with acyl residue C16:1 | No | No | ↑ | < 0.05 |
| Phosphatidylcholine with acyl-alkyl residue sum C26:0 | No | No | ↑ | < 0.03 |
| Phosphatidylcholine with acyl-alkyl residue sum C30:0 | ↓ | No | No | < 0.05 |
| Phosphatidylcholine with acyl-alkyl residue sum C32:0 | ↓ | No | No | < 0.04 |
| Phosphatidylcholine with acyl-alkyl residue sum C32:1 | No | ↓ | No | < 0.05 |
| Phosphatidylcholine with acyl-alkyl residue sum C36:0 | No | No | ↓ | < 0.04 |
| Phosphatidylcholine with acyl-alkyl residue sum C36:5 | No | No | ↓ | < 0.02 |
| Phosphatidylcholine with acyl-alkyl residue sum C38:0 | No | No | ↓ | < 0.02 |
| Phosphatidylcholine with acyl-alkyl residue sum C38:1 | No | No | ↓ | < 0.01 |
| Phosphatidylcholine with acyl-alkyl residue sum C38:5 | No | No | ↓ | < 0.03 |
| Phosphatidylcholine with acyl-alkyl residue sum C40:1 | No | No | ↓ | < 0.02 |
| Phosphatidylcholine with acyl-alkyl residue sum C40:5 | No | No | ↓ | < 0.01 |
| Phosphatidylcholine with acyl-alkyl residue sum C42:0 | No | No | ↓ | < 0.01 |
| Phosphatidylcholine with acyl-alkyl residue sum C42:1 | No | No | ↓ | < 0.05 |
| Phosphatidylcholine with acyl-alkyl residue sum C42:5 | No | No | ↓ | < 0.03 |
| Phosphatidylcholine with acyl-alkyl residue sum C42:6 | No | No | ↓ | < 0.03 |
| Phosphatidylcholine with diacyl residue sum C32:1 | ↓ | ↓ | No | < 0.04 |
| Phosphatidylcholine with diacyl residue sum C32:2 | No | No | ↓ | < 0.02 |
| Phosphatidylcholine with diacyl residue sum C36:0 | No | No | ↓ | < 0.01 |
| Phosphatidylcholine with diacyl residue sum C36:4 | ↓ | ↓ | ↓ | < 0.04 |
| Phosphatidylcholine with diacyl residue sum C36:5 | ↓ | ↓ | ↓ | < 0.04 |
| Phosphatidylcholine with diacyl residue sum C38:0 | No | No | ↓ | < 0.05 |
| Phosphatidylcholine with diacyl residue sum C38:1 | No | No | ↓ | < 0.05 |
| Phosphatidylcholine with diacyl residue sum C38:1 | No | No | ↓ | < 0.03 |
| Phosphatidylcholine with diacyl residue sum C38:2 | No | No | ↓ | < 0.03 |
| Phosphatidylcholine with diacyl residue sum C38:3 | No | No | ↓ | < 0.02 |
| Phosphatidylcholine with diacyl residue sum C38:4 | No | ↓ | ↓ | < 0.04 |
| Phosphatidylcholine with diacyl residue sum C38:5 | No | ↓ | ↓ | < 0.03 |
| Phosphatidylcholine with diacyl residue sum C38:6 | No | No | ↓ | < 0.04 |
| Phosphatidylcholine with diacyl residue sum C40:1 | No | No | ↓ | < 0.04 |
| Phosphatidylcholine with diacyl residue sum C40:2 | No | No | ↓ | < 0.02 |
| Phosphatidylcholine with diacyl residue sum C40:4 | No | No | ↓ | < 0.02 |
| Phosphatidylcholine with diacyl residue sum C40:6 | No | No | ↓ | < 0.02 |
| Phosphatidylcholine with diacyl residue sum C42:3 | No | No | ↓ | < 0.03 |
| Phosphatidylcholine with diacyl residue sum C42:4 | No | No | ↓ | < 0.02 |
| Phosphatidylcholine with diacyl residue sum C42:5 | No | No | ↓ | < 0.05 |
| Phosphatidylcholine with diacyl residue sum C44:3 | No | No | ↓ | < 0.02 |
| Phosphatidylcholine with diacyl residue sum C44:5 | No | No | ↓ | < 0.02 |
| Phosphatidylcholine with diacyl residue sum C44:6 | No | No | ↓ | < 0.01 |
| PUFA (PC) | No | No | ↓ | < 0.01 |
| SFA (PC) | No | No | ↓ | < 0.03 |
| Total (PC+SM) | No | No | ↓ | < 0.02 |
| Total lysoPC / Total PC | ↑ | ↑ | ↑ | < 0.02 |
| Total Phosphatidylcholine | No | No | ↓ | < 0.01 |
| Hydroxysphingomyelin with acyl residue sum C14:1 | ↓ | No | No | < 0.02 |
| Hydroxysphingomyelin with acyl residue sum C22:2 | No | No | ↓ | < 0.02 |
| Hydroxysphingomyelin with acyl residue sum C18:1 | ↓ | ↓ | ↓ | < 0.02 |
| Hydroxysphingomyelin with acyl residue sum C20:2 | No | ↓ | No | < 0.02 |
| Hydroxysphingomyelin with acyl residue sum C22:3 | No | No | ↓ | < 0.02 |
| Acylcarnitines-C12:1 | No | ↓ | ↓ | < 0.03 |
| Acylcarnitines-C14:1 | No | ↓ | ↓ | < 0.01 |
| Acylcarnitines-C14:2 | No | No | ↓ | < 0.03 |
| **LC compounds** |  |  |  |  |
| Alanine | No | No | ↑ | <0.05 |

Additional file 1: Figure S10. Survival curves of the fish following 9 hours bath exposure to 1.6 × 10^7^ CFU·mL^-1^ of live *Y. ruckeri* O1 biotype 2 (100415-1/4).


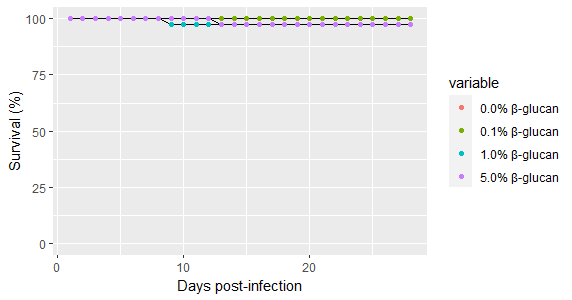

Supplement: Supplementary file 1 — Additional file 1: Table S1. Allocation of the fish at the start of the experiment. Table S2. Analysis of the composition of the β-glucan used in this study. Figure S3. Principal component analysis showing the microbiota of the fish at week 6. Figure S4. Heatmap showing the 10 most abundant bacterial phyla in the intestine of the fish for the two control groups and the groups receiving the three different concentration of β-glucan. Table S5. Performance of the fish over the course of the experiment. Figure S6. Average weight of the fish per treatment over the course of the experiment. Figure S7. Average Fulton's condition factor (K) of the fish per treatment over the course of the experiment. Table S8. All metabolites measured in the serum of the fish for all investigated groups. Table S9. Metabolites measured at significantly different levels between treated fish and the control. Arrows indicate if the metabolite were upregulated (↑) or downregulated (↓) relative to the control without β-glucan treatment. Lack of significant change is indicated by “No”. Figure S10. Survival curves of the fish following 9 h bath exposure to 1.6 × 10^7^ CFU∙mL−1 of live Y. ruckeri O1 biotype 2 (100415-1/4). [file 42523_2022_209_MOESM1_ESM.docx]
